# Brain co‐delivery of first‐line chemotherapy drug and epigenetic bromodomain inhibitor for multidimensional enhanced synergistic glioblastoma therapy

**DOI:** 10.1002/EXP.20210274

**Published:** 2022-04-19

**Authors:** Yanjie Liu, Wendie Wang, Dongya Zhang, Yajing Sun, Fangzhou Li, Meng Zheng, David B. Lovejoy, Yan Zou, Bingyang Shi

**Affiliations:** ^1^ Henan–Macquarie University Joint Centre for Biomedical Innovation Academy for Advanced Interdisciplinary Studies Henan Key Laboratory of Brain Targeted Bio‐nanomedicine School of Life Sciences Henan University Kaifeng Henan China; ^2^ CAS Key Laboratory for Biomedical Effects of Nanomaterials and Nanosafety CAS Center for Excellence in Nanoscience National Center for Nanoscience and Technology of China Beijing China; ^3^ Centre for Motor Neuron Disease Research Macquarie Medical School Faculty of Medicine, Health and Human Sciences Macquarie University Sydney New South Wales Australia

**Keywords:** biomimetic, blood–brain barrier, brain‐targeted delivery, chemoimmunotherapy, glioblastoma

## Abstract

Glioblastoma (GBM) is a central nervous system tumor with poor prognosis due to the rapid development of resistance to mono chemotherapy and poor brain targeted delivery. Chemoimmunotherapy (CIT) combines chemotherapy drugs with activators of innate immunity that hold great promise for GBM synergistic therapy. Herein, we chose temozolomide, TMZ, and the epigenetic bromodomain inhibitor, OTX015, and further co‐encapsulated them within our well‐established erythrocyte membrane camouflaged nanoparticle to yield ApoE peptide decorated biomimetic nanomedicine (ABNM@TMZ/OTX). Our nanoplatform successfully addressed the limitations in brain‐targeted drug co‐delivery, and simultaneously achieved multidimensional enhanced GBM synergistic CIT. In mice bearing orthotopic GL261 GBM, treatment with ABNM@TMZ/OTX resulted in marked tumor inhibition and greatly extended survival time with little side effects. The pronounced GBM treatment efficacy can be ascribed to three key factors: (i) improved nanoparticle‐mediated GBM targeting delivery of therapeutic agents by greatly enhanced blood circulation time and blood–brain barrier penetration; (ii) inhibited cellular DNA repair and enhanced TMZ sensitivity to tumor cells; (iii) enhanced anti‐tumor immune responses by inducing immunogenic cell death and inhibiting PD‐1/PD‐L1 conjugation leading to enhanced expression of CD4^+^ and CD8^+^ T cells. The study validated a biomimetic nanomedicine to yield a potential new treatment for GBM.

## Graphical abstract

We developed temozolomide and epigenetic bromodomain inhibitor co‐encapsulated biomimetic nanomedicine (ABNM@TMZ/OTX) achieved multidimensional enhanced glioblastoma synergistic chemoimmunotherapy in both primary and recurrent orthotopic mice models with significant extended survival rate and little side effects.

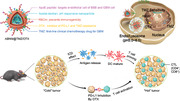

## 1 INTRODUCTION

Glioblastoma multiforme (GBM) is the most lethal central nervous system tumor due to its diffuse infiltrative nature which results in incomplete surgical resection and the development of drug resistance.^[^ ^1^ ^]^ In the past decade, the median survival time of GBM patients has not improved significantly, with 5 year‐life expectancy being lower than 10%.^[^ ^2^ ^]^ The standard of care for patients with malignant GBM is postoperative treatment with temozolomide (TMZ) adjuvant to radiation.^[^ ^3^ ^]^ Unfortunately, the effectiveness of this treatment regimen remains low, in part, due to the limitations of drug monotherapy that leads to rapid development of drug resistance.^[^ ^4^ ^]^ Additional limitations of TMZ include limited blood–brain barrier (BBB) permeability and poor accumulation in tumor.^[^ ^5^ ^]^

As the pathogenic mechanisms of GBM are complex,^[^ ^6^ ^]^ combinatorial therapy that targets multiple oncogenic molecular pathways has a better chance of avoiding the development of drug resistance associated with monotherapies. Recently, treatments that modulate immune‐regulation have attracted intensive attention,^[^ ^7^ ^]^ strategies such as cancer vaccines,^[^ ^8^ ^]^ immune checkpoint blockade immunotherapy,^[^ ^9^ ^]^ and adoptive cell therapy (e.g., CAR‐T),^[^ ^10^ ^]^ have shown encouraging clinical results in treating various types of cancers including GBM. Among them, blockade therapy using immune checkpoint inhibitors targeting the programmed death‐1 (PD‐1)/programmed death‐ligand 1 (PD‐L1) pathway, has become a first option for many cancers and has significantly changed the landscape of cancer therapy.^[^ ^11^ ^]^ However, the commercial anti‐PD‐1 antibodies, nivolumab, and pembrolizumab showed limited therapeutic effects in GBM patients with less than 10% of patients showing long‐term responses,^[^ ^12^ ^]^ attributable to tumors remaining “cold” and immunosuppressive as characterized by the high levels of immunosuppressive cytokines, low mutation rate, and relatively poor tumor T cell infiltration.^[^ ^13^ ^]^ OTX015 (OTX) is an unique small molecule agent (a bromodomain‐containing protein 4 (BRD4) inhibitor) which can turn the so‐called “cold” immunosuppressive GBM into T cell‐inflamed “hot” tumor by inhibiting tumor expression of PD‐L1,^[^ ^14^ ^]^ leading to effective immunotherapy. Moreover, OTX is able to interfere with cell proliferation by inducing cell cycle arrest.^[^ ^15^ ^]^ Importantly, OTX potentiates tumor sensitivity to TMZ by reducing the DNA damage repair response during cell cycle disruption,^[^ ^16^ ^]^ thus helping to overcome TMZ resistance in GBM. These features make OTX an ideal therapeutic partner with TMZ to generate synergistic GBM therapy from different dimensions.

**FIGURE 1:**
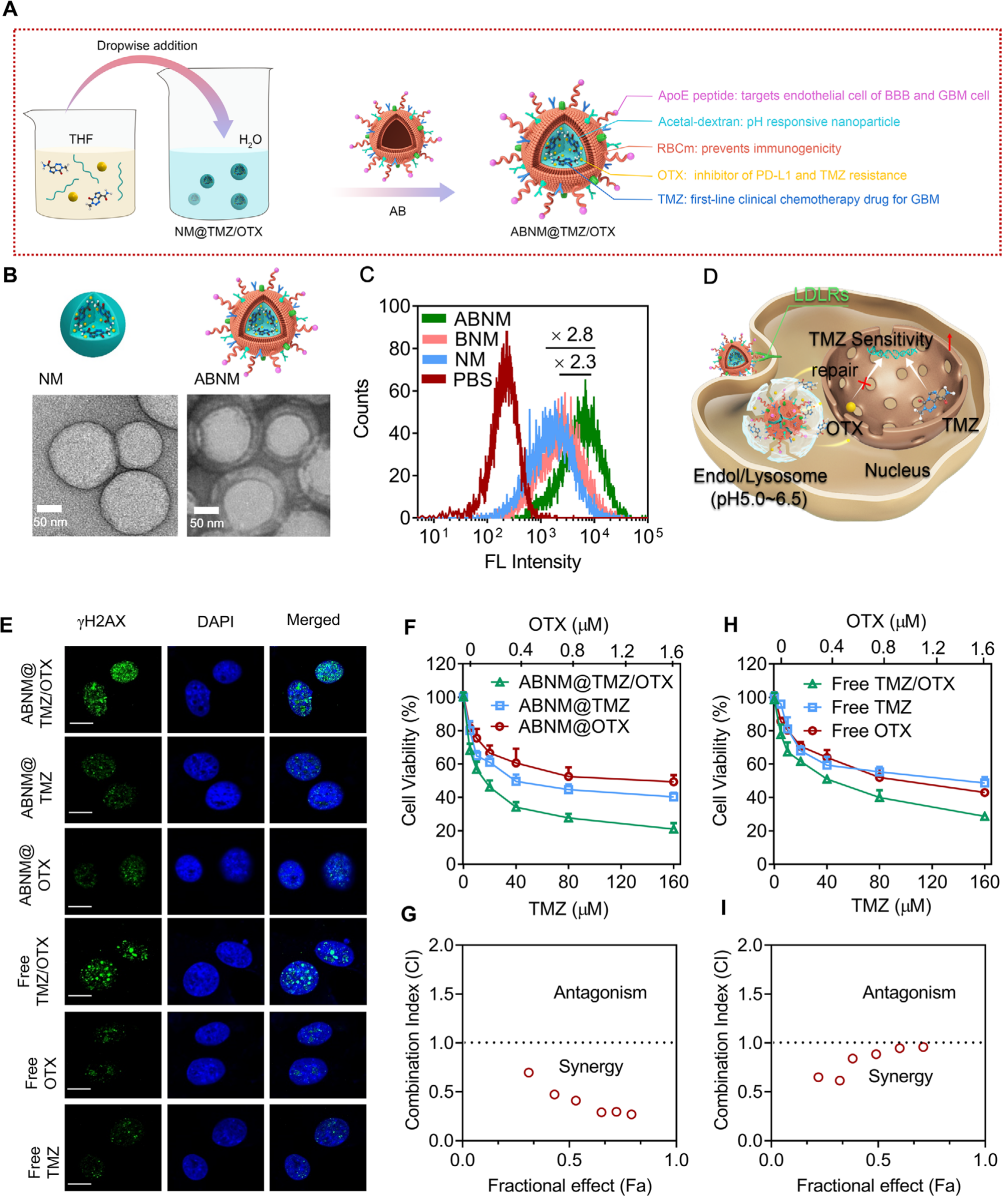
Fabrication, cell uptake, and combinational therapy effects of AMNM@TMZ/OTX. (A) ABNM@TMZ/OTX nanomedicine was generated from AopE peptide modified red blood cell membrane, TMZ, and OTX co‐loaded pH‐sensitive nanomedicines. (B) TEM images of the naked nanomedicine core (NM, left) and ApoE decorated biomimetic nanomedicine (ABNM, right). (C) Flow cytometry of GL261 cells treated with 4 h incubation with ABNM (FITC tagged nanoparticles, FITC: 0.5 μg/mL). (D) Schematic illustration of TMZ chemo‐immunotherapy mediated by ABNM@TMZ/OTX against GL261 GBM cells. TMZ kills GBM tumor cells, co‐delivery of OTX inhibits cell proliferation, limits DNA damage repair, and potentiates TMZ sensitivity. (E) DNA damage foci of GL261 cells receiving ABNM@TMZ/OTX, ABNM@TMZ, ABNM@OTX, free TMZ/OTX, free OTX, and free TMZ for 72 h (TMZ: 150 μM; OTX: 400 nM). Scale bar = 10 μm. (F) Cell proliferation studies in GL261 GBM cells after 72 h incubation with ABNM@TMZ/OTX, ABNM@TMZ, or ABNM@OTX. (G) The Chou‐Talalay Fa‐CI plot of ABNM@TMZ/OTX treatment. (H) Cell proliferation studies in GL261 GBM cells after 72 h incubation with free TMZ/OTX, free TMZ, or free OTX. (I) The Chou‐Talalay Fa‐CI plot of free TMZ/OTX treatment

Given that OTX has limited BBB penetration and poor GBM targeting as well,^[^ ^17^ ^]^ we co‐encapsulated TMZ together with OTX using the well‐established cell membrane coating approaches in our lab^[^ ^18^ ^]^ to improve brain‐targeted codelivery of TMZ+OTX and GBM targeting (Figure 1A). By design, externally, our nanomedicine is cloaked with red blood cell membrane (RBCm) decorated with Apolipoprotein E peptide (ApoE) to promote BBB permeability and GBM cellular uptake.^[^ ^19^ ^]^ Internally, we used a pH‐responsive polymer that degrades in low pH to promote GBM micro‐ and intracellular environment triggered drug release. Together, these design elements generate ABNM@TMZ/OTX nanomedicine and we hypothesized that ABNM@TMZ/OTX should realize high‐performance synergistic GBM therapy. To verify our hypothesis, we firstly characterized ABNM@TMZ/OTX nanomedicine and evaluated its cell targeting and synergistic therapeutic effect in vitro; we then assessed its immune response, BBB penetration, and GBM targeting capabilities in vivo. Next, we systematically evaluated the synergistic therapeutic effectiveness using GL261 GBM bearing mice and a tumor‐recurrence GBM mouse model to simulate practical clinical therapy. Lastly, we comprehensively assessed the safety profile of the ABNM@TMZ/OTX nanomedicine.

## 2 RESULTS

### 2.1 Preparation and characterization of ABNM@TMZ/OTX

The biomimetic nanomedicine ABNM@TMZ/OTX was fabricated according to our previous work.^[^ ^18^ ^]^ As illustrated in Figure 1A, the inner core was comprised of TMZ and OTX encapsulated pH‐sensitive nanoparticles via self‐assembling and further coated with ApoE decorated red blood cell membrane (ApoE­RBCm) to obtain the biomimetic nanomedicine ABNM@TMZ/OTX. The TMZ and OTX loading capacity was determined by high performance liquid chromatography (HPLC, Agilent 1260) which showed a high TMZ and OTX loading content of 6.7% and 7.6%, respectively (Table S1). RBCm vesicles from RBCs were functionalized by incorporating ApoE peptide as a targeting ligand following our previous protocol to obtain ApoE­RBCm.^[^ ^18^ ^]^ The ApoE peptide can specifically bind to the low‐density lipoprotein receptor family (LDLRs) highly expressed on both brain endothelial and tumor cells, leading to the “two birds, one stone” targeting strategy where ApoE will drive ABNM@TMZ/OTX to pass the BBB firstly and then target the tumor cells in brain. A sonication method was used to coat ApoE­RBCm onto the surface of the bare nanomedicine NM@TMZ/OTX to acquire the final biomimetic nanomedicine ABNM@TMZ/OTX. The obtained nanomedicines had a size of 186 nm (Table S1 and Figure S1), which was 18 nm larger than bare nanomedicine (168 nm) and consistent with the thickness of RBC membranes.^[^ ^20^ ^]^ We used transmission electron microscopy (TEM) imaging to further confirm the completeness of the ABNM@TMZ/OTX core‐shell structure (Figure 1B). In vitro drug release results showed that TMZ release was triggered from ABNM@TMZ/OTX under acidic conditions, with 67% and 45% TMZ release within 24 h at pH 5.0 and 6.5, respectively (Figure S2A). In sharp contrast, our nanomedicine remained stable under physiological conditions less than 20% released within 24 h. OTX release from ABNM@TMZ/OTX exhibited a similar kinetic profile (Figure S2B), indicating the fast pH responsiveness of these nanomedicines and showing that RBCm modification had little impact on stimuli‐triggered TMZ/OTX release.

### 2.2 Receptor‐mediated cellular uptake in GBM tumor

Initially, we identified whether LDLRs (LDL receptor (LDLR) and LDLR‐related protein 1 (LRP1) are over‐expressed in GL261 GBM cells by western blotting assays in GL261 cells and HA1800 astroglia. The results showed that GL261 cells over‐expressed LRP1 and LDLR, while HA 1800 astroglia weakly expressed these proteins (Figure S3). Hence, ApoE peptide functionalization could be specifically recognized by LDLR and LRP1 highly expressing cells and would further be a feasible method to modify nanomedicines to achieve high GL261 cells targeting ability.

Accordingly, LDLRs‐mediated GL261 cellular uptake and intracellular drug release were studied by flow cytometry and confocal laser scanning microscopy (CLSM) using FITC‐labeled ABNM. ABNM showed approximately 2.3‐fold enhanced FITC fluorescence intensity compared to BNM (non‐targeted control) (Figure 1C). In addition, pronounced FITC fluorescence was observed in the cytoplasm of GL261 cells after incubation with ABNM for 4 h, which was much stronger than that of BNM or bare NM (Figure S4), indicating the efficient internalization and active targeting capability of biomimetic nanomedicines via receptor mediated endocytosis. Interestingly, ABNM further exhibited an enhanced cellular uptake toward bEnd3 endothelial cells (Figure S5), laying a fundamental to cross blood–brain barrier (BBB). The in vitro BBB transwell study showed that the ABNM displayed a significantly high transport ratio as compared with non‐targeting control (Figure S6), emphasizing the ApoE decorated ABNM possessing efficient BBB permeability.

### 2.3 TMZ and OTX combinational therapy in GL261 cells

As gliomas are liable to develop drug resistance to TMZ in clinic, we studied whether co‐delivery of a BRD4 inhibitor (OTX) could suppress the tumor cells and re‐sensitize cells to TMZ (Figure 1D). At first, we treated GL261 cells with ABNM@TMZ/OTX for 72 h. The DNA damage signal of γH2AX was observed in cells which also resulted from treatment with ABNM@TMZ loaded with 150 μM TMZ. Importantly, synergistic effects appeared when cells were incubated with OTX and TMZ co‐encapsulated ABNM@TMZ/OTX (Figure 1E). Next, we further examined the combinatorial effects of co‐encapsulation of TMZ and OTX, we performed cell viability assays in GL261 cells and the half‐maximal inhibitory concentration (IC_50_) was examined at various combinations of TMZ and OTX. Treatment with both nanomedicines or free drugs significantly lowered the baseline IC_50_ of TMZ illustrating that OTX enhanced the sensitivity of cells to TMZ (Figure 1F,H). Examining Chou‐Talalay synergistic effects showed that ABNM@TMZ/OTX nanomedicine exhibited greater synergy (lower combination index (CI) value; Figure 1G) than free drugs (Figure 1I), with the strongest CI value being 0.2 in the GL261 cell for ABNM@TMZ/OTX. Of note, the different trends of CI values between free drugs and nanomedicines mainly attribute to the distinct cell uptake and intracellular drug release kinetics. These data confirm that OTX enhances the cytotoxicity of TMZ, which is consistent with our observation that co‐delivery of OTX and TMZ enhanced DNA damage compared to either drug alone.

### 2.4 Immunogenic cell death induced by ABNM@TMZ/OTX

Well‐known hallmarks of immunogenic cell death (ICD) are calreticulin (CRT) translocation, nonhistone chromatin protein (HMGB1) release, and adenosine triphosphate (ATP) secretion.^[^ ^21^ ^]^ Accordingly, we determined the effect of TMZ and OTX co‐loaded ABNM@TMZ/OTX nanomedicines on CRT and HMGB1 expression by immunofluorescence in GL261 cell. Cell membrane expression of CRT was maximally increased by ABNM@TMZ/OTX and a lesser extent induced by ABNM@OTX and ABNM@TMZ (Figure S7). HMGB1 release followed the same tendency (Figure S8). ATP release in GL261 cells again was maximal after treatment with ABNM@TMZ/OTX relative to nanomedicines containing mono‐drugs or treatment with free drugs (Figure S9). Collectively, these results highlight the ability of ABNM@TMZ/OTX to induce tumor ICD, which may have potential as an immune‐based therapy.

### 2.5 Reduction of PD‐L1 expression induced by ABNM@TMZ/OTX

The expression of PD‐L1 of tumor cell is associated with immune escape,^[^ ^22^ ^]^ which can bind to the PD‐1 expressed of tumor‐infiltrating cytotoxic T‐cells (CTLs). We detected the PD‐L1 expression by flow cytometry and the results showed that ABNM@TMZ/OTX completely reversed PD‐L1 expression in GL261 cells by inhibiting BRD4 activation (Figure S10), supporting the pronounced PD‐L1 inhibition mediated by ABNM@TMZ/OTX.

### 2.6 Immune‐stimulation by ABNM@TMZ/OTX

**FIGURE 2:**
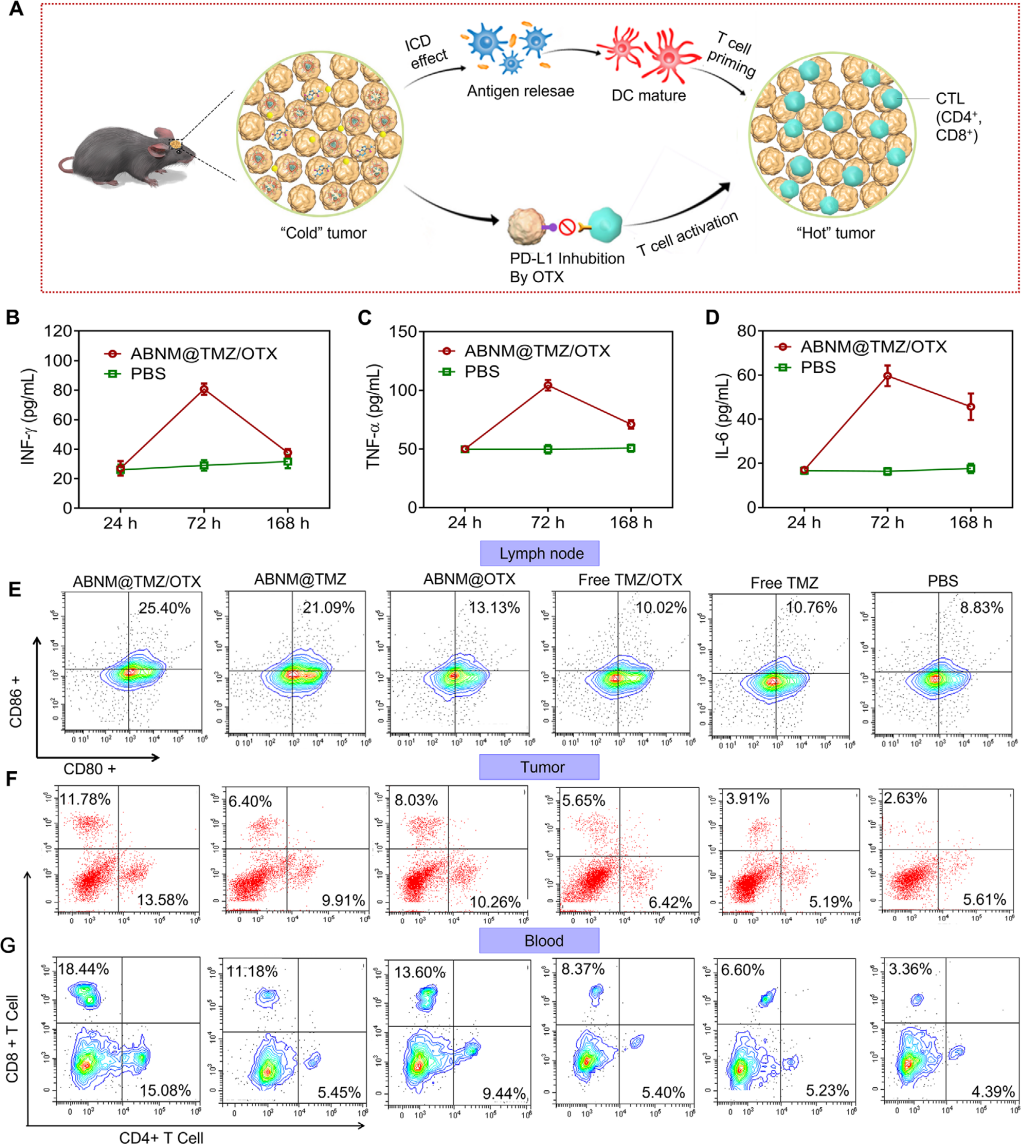
Antitumor immunity enhanced by biomimetic nanomedicine ABNM@TMZ/OTX. (A) Schematic illustration of antitumor immune response and enhanced chemo‐immunotherapy induced by ABNM@TMZ/OTX against GL261 cells. TMZ chemotherapy activates innate antitumor immune responses via induction of ICD, co‐delivery of OTX suppresses the expression of PD‐L1, promotes tumor T cell activation, induces antitumor immune responses, turns “cold” GBM into “hot” tumor, and achieves combinational tumor therapy. Serum concentrations of (B) IFN‐γ, (C) TNF‐α, and (D) IL‐6 at 24, 72, and 168 h post treatment. Treatment with ABNM@TMZ/OTX induced antitumor immunity in vivo. (E) Activated dendritic cells (DCs) in tumor‐draining lymph nodes in mice treated with ABNM@TMZ/OTX, ABNM@TMZ, ABNM@OTX, free TMZ/OTX, or free TMZ. A single dose of 5.0 mg TMZ equiv./kg and/or 5.0 mg OTX equiv./kg was intravenously injected into tumor bearing mice. Following sacrifice 72 h after treatment, anti‐80‐PE and anti‐CD86‐APC were used to stain CD80 and CD86 markers in DCs. Mice treated with PBS were used as controls (n = 3). Infiltration of CD4^+^ and CD8^+^ T cells in tumor (F) and blood (G) by flow cytometry which were taken from GL261‐Luc mice 72 h post injection treated with ABNM@TMZ/OTX (5 mg TMZ equiv. kg^−1^, 5 mg OTX equiv. kg^−1^)

We further investigated the ability of ABNM@TMZ/OTX nanomedicine to induce immune‐stimulation in vivo in C57BL/6 immunocompetent mice bearing orthotopic GL261 tumor (Figure 2A). After a single administration, levels of INF‐γ, TNF‐α, and IL‐6 in the peripheral serum, were 2.8‐, 2.1‐ and 3.7‐fold higher than that of mice receiving PBS (72 h post‐injection; Figure 2B–D). As the co‐stimulatory molecules CD80 and CD86 are widely used as biomarkers of dendritic cells (DCs) maturation,^[^ ^23^ ^]^ we investigated the immunoregulatory effect on DCs after ABNM@TMZ/OTX treatment. In mice treated with ABNM@TMZ/OTX, expression of CD80 and CD86 in lymph nodes increased to 25.40% compared to 8.83% in mice receiving PBS. Mice receiving ABNM@TMZ or ABNM@OTX mono‐drug loaded nanomedicines showed reduced CD80 and CD86 expression at 21.09% and 13.13%, respectively (Figure 2E). Interestingly, free TMZ/OTX mixture or free TMZ did not significantly induce DCs compared to PBS treatment, which may be ascribed to the unfavorable pharmacokinetics of free drugs. These results suggest that ABNM@TMZ/OTX biomimetic nanomedicine induces a strong immune response, demonstrating its potential as an immunotherapeutic agent.

To better understand the checkpoint blockade process induced by our ABNM@TMZ/OTX immunotherapy, we assessed tumor‐infiltrating CTLs profile in tumor and blood with flow cytometry. Cytotoxic CD8^+^ T cells and CD4^+^ T helper cells play key roles in adaptive immunity with increases in CD8^+^ and CD4^+^ promoting cancer immunotherapy. The total percentage of CD8^+^ and CD4^+^ T cells in tumors from mice treated with ABNM@TMZ/OTX was 25.36%, which was 2.1‐times higher than that seen in mice treated with free TMZ/OTX (Figure 2F). Similarly, ABNM@TMZ/OTX treatment induced the highest levels of CD8^+^ and CD4^+^ in blood (Figure 2G). Collectively, these results indicate that our nanomedicine delivery strategy successfully enhances tumor‐infiltrating lymphocytes.

### 2.7 Enhanced circulation and targeted brain delivery of TMZ and OTX mediated by ABNM@TMZ/OTX

**FIGURE 3:**
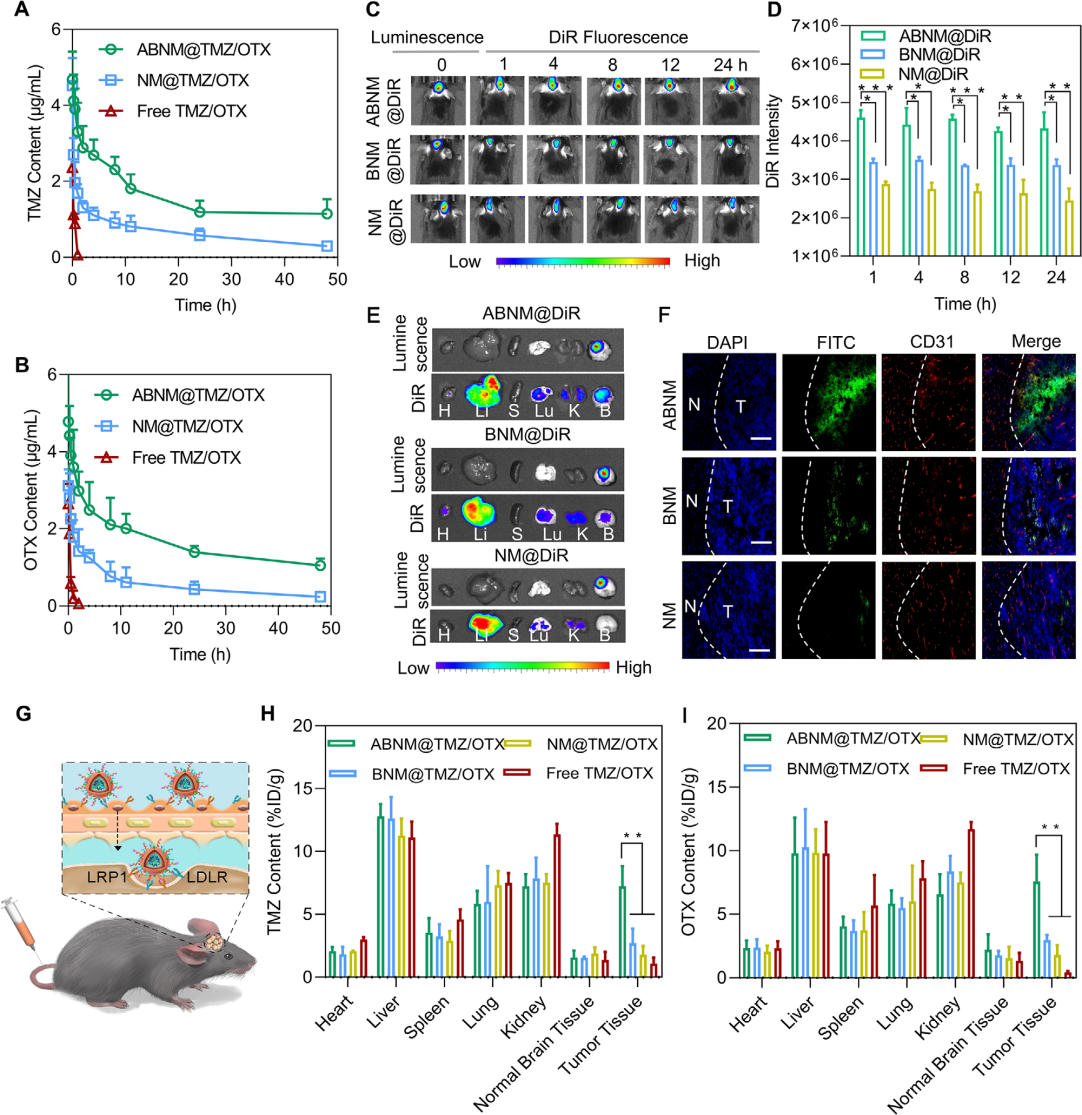
Pharmacokinetics, in vivo imaging, BBB penetration, and biodistribution. Quantification of (A) TMZ and (B) OTX in mice receiving ABNM@TMZ/OTX, NM@TMZ/OTX or free TMZ/OTX (5 mg TMZ equiv./kg, 5 mg OTX equiv./kg) post administration. (C) In vivo fluorescence images and (D) DiR quantitative results of GL261‐Luc orthotopic mice at different time points after treatment with ABNM@DiR, BNM@DiR, or NM@DiR (1 mg DiR equiv. kg^−1^). (E) DiR fluorescence images of the major organs from C57BL/6 mice bearing orthotopic GL261‐Luc tumor 8 h after receiving ABNM@DiR, BNM@DiR, or NM@DiR (1 mg DiR equiv./kg). (F) Tumor penetration behavior of FITC‐labeled ABNM observed by CLSM. Dotted lines indicate tumor boundary. N: normal brain tissue, T: tumor tissue. The scale bars correspond to 50 μm. (G) Schematic illustration of BBB penetration by ABNM@TMZ/OTX via receptor mediated transcytosis. Quantification of (H) TMZ and (I) OTX distribution in major organs and tumor from C57BL/6 mice bearing orthotopic GL261‐Luc after i.v. injection of ABNM@TMZ/OTX, BNM@TMZ/OTX, NM@TMZ/OTX and free TMZ/OTX (5 mg TMZ equiv./kg, 5 mg OTX equiv./kg)

ABNM@TMZ/OTX exhibited remarkably extended blood retention time over a span of 48 h with an elimination half‐life time (t_1/2,β_) of 7.2 h for TMZ (Figure 3A). However, the t_1/2,β_ of uncoated group (NM@TMZ/OTX) is 2.3 h, which revealed that the strategy of decorating RBCm successfully prolonged blood circulation time of ABNM@TMZ/OTX. Interestingly, RBCm coated nanomedicines BNM@TMZ/OTX without peptide may have similar pharmacokinetic profile with that of ABNM@TMZ/OTX as the targeting ligand have negligible effects on the blood circulation as observed in our previous work.^[^ ^18b^ ^]^ ABNM@TMZ/OTX led to similarly increased blood circulation of TMZ and OTX (Figure 3B), highlighting the ability of ABNM@TMZ/OTX to potentiate systemic exposure.

The in vivo tumor targeting ability of ABNM@TMZ/OTX was then evaluated in C57BL/6 mice bearing orthotopic luciferase stable‐tagged GL261 (GL261‐Luc) tumors by intravenous injection of nanomedicines. A near‐infrared dye, DiR, was loaded into nanomedicines to monitor biodistribution. At 1 h post injection of ABNM@DiR, the fluorescence of DiR was observed in the brain site and the maximum fluorescence intensity was reached at 8 h, and the high fluorescence was maintained up to 24 h (Figure 3C). The DiR quantitative results further corroborated the active targeting ability of ABNM@DiR (Figure 3D). These data confirm the important role of ApoE peptide in promoting BBB traversal, active targeting, and brain accumulation.

To further confirm the role of ApoE peptide, ex vivo fluorescence of the major organs illustrated that mice treated with ABNM@DiR had higher DiR fluorescence in orthotopic tumor compared to heart, spleen, lung, and kidney. However, less DiR fluorescence was observed in orthotopic brain tumors of mice injected with BNM@DiR and NM@DiR (Figure 3E). Remarkably, strong fluorescence was observed in both liver and kidney for all the groups, that's mainly attributed to the fact that nanomedicines are eliminated from body by reticuloendothelial system that consists of tissues including liver and kidney.^[^ ^24^ ^]^ Tumor penetration studies indicated that ABNM had a higher accumulation in blood vessels and tumors due to active targeting ability of ABNM (Figure 3F). Interestingly, deep glioma penetration was also observed, and fluorescence was accumulated throughout the whole tumor region with little distribution in normal brain area, indicating the effective and specific GBM targeting ability of ABNM via receptor‐mediated transcytosis mechanism (Figure 3G).

We next examined drug delivery in mice bearing GL261‐Luc GBM tumor treated with ABNM@TMZ/OTX. The level of TMZ in GBM tumor reached 7.22% injected dose (ID)/g, which was comparable to liver and kidney but markedly higher than other organs including heart, spleen, lung, and normal brain tissue (Figure 3H). In contrast, much lower tumor accumulation of TMZ was observed for NM@TMZ/OTX and free TMZ/OTX, where TMZ level was 1.37%‐ and 0.54% ID/g, respectively. OTX showed a similar accumulation profile in GBM tumor and major tissues as TMZ (Figure 3I). The improved accumulation of ABNM@TMZ/OTX in GBM most likely reflects enhanced BBB permeability and specific GBM targeting.

### 2.8 Superior therapeutic efficacy of ABNM@TMZ/OTX

**FIGURE 4:**
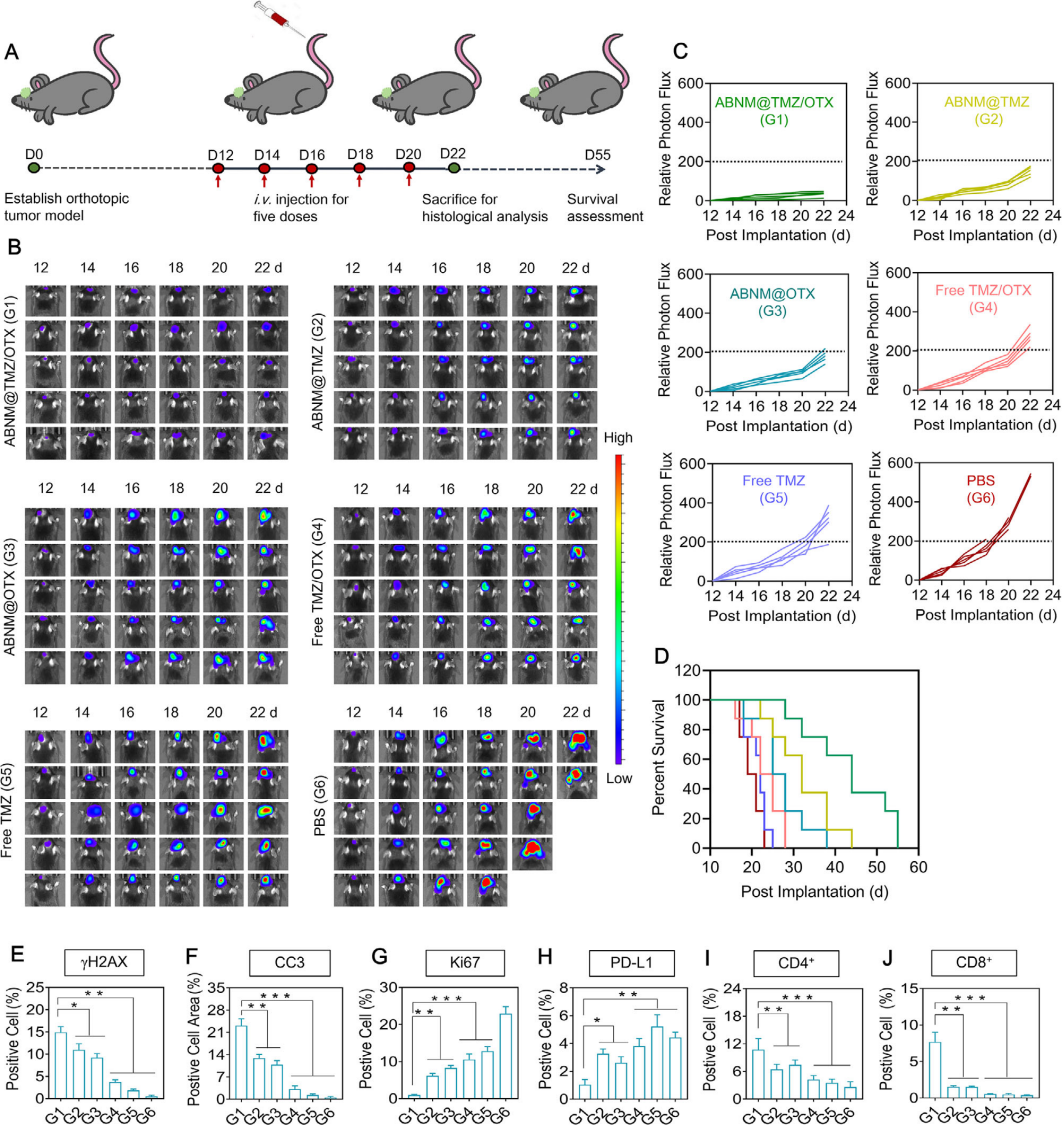
Enhanced anti‐tumor effects mediated by ABNM@TMZ/OTX. (A) Schematic timeline of the anti‐tumor efficacy study. (B) Bioluminescent signal images of mice bearing orthotopic GL261‐Luc GBM tumor after receiving ABNM@TMZ/OTX, ABNM@TMZ, ABNM@OTX, free TMZ/OTX, free TMZ, or PBS. Mice were intravenously injected at a dose of 5.0 mg TMZ equiv./kg and/or 5.0 mg OTX equiv./kg on days 12, 14, 16, 18, and 20 post‐tumor implantation. (C) Quantified luminescence levels of tumor in mice as treated in b). (D) Mice survival rates. Quantification of number of tumor cells that stained positive for markers of (E) γH2AX, (F) CC3, (G) Ki67, (H) PD‐L1, (I) CD4^+^, and (J) CD8^+^

The anti‐tumor efficacy of ABNM@TMZ/OTX was examined in GL261‐Luc GBM bearing C57BL/6 mice 12 days post intracranial implantation by intravenous injection of nanomedicine containing 5 mg TMZ equiv. kg^–1^ and 5 mg OTX equiv. kg^–1^, every 2 days (Figure 4A). Treatment with ABNM@TMZ/OTX showed the most effective tumor growth suppression as reflected by the lowest bioluminescence among all the treatment groups (Figure 4B,C). Importantly, treatment with ABNM@TMZ/OTX significantly prolonged survival of GL261 mice compared to mice treated with either free drug or mono‐drug loaded nanomedicines (Figure 4D). The median survival time of mice following ABNM@TMZ/OTX treatment was 44 days, significantly longer than that of ABNM@TMZ (32 days), ABNM@OTX (26.5 days), free TMZ/OTX (23.5 days), free TMZ (22 days) or PBS (20 days). Major organs, including liver and kidney, taken from mice treated with ABNM@TMZ/OTX exhibited no obvious signs of toxicity compared to PBS treatment. However, the free drug combination and free TMZ induced minor renal toxicity (Figure S11). Furthermore, quantification of immuno‐fluorescence and immunohistochemistry (IHC) images showed that ABNM@TMZ/OTX exhibited the highest level of DNA damage markers (γH2AX; Figure 4E) and tumor cell apoptosis (TUNEL, CC3; Figure 4F, Figure S12). Additionally, ABNM@TMZ/OTX treatment induced the lowest level of the tumor cell proliferation marker Ki67 in tumor slides (Figure 4G). Importantly, considerable reductions in PD‐L1 expression were seen in tumor slices from mice treated with ABNM@TMZ/OTX (Figure 4H), supporting the efficient modulation of PD‐L1 by nanomedicine‐delivered OTX. Moreover, numbers of cytotoxic CD8^+^ T and CD4^+^ T helper cells were significantly enhanced compared to other treatments, further confirming innate immune activation by ABNM@TMZ/OTX (Figure 4I,J). All these results demonstrate that the superior tumor inhibition of ABNM@TMZ/OTX is benefited from the co‐delivery of OTX, which not only caused the anti‐tumor immune response but also enhanced the sensitivity of GBM tumor cells to TMZ.

### 2.9 Combinational therapy by ABNM@TMZ/OTX in a recurrence model of GBM

**FIGURE 5:**
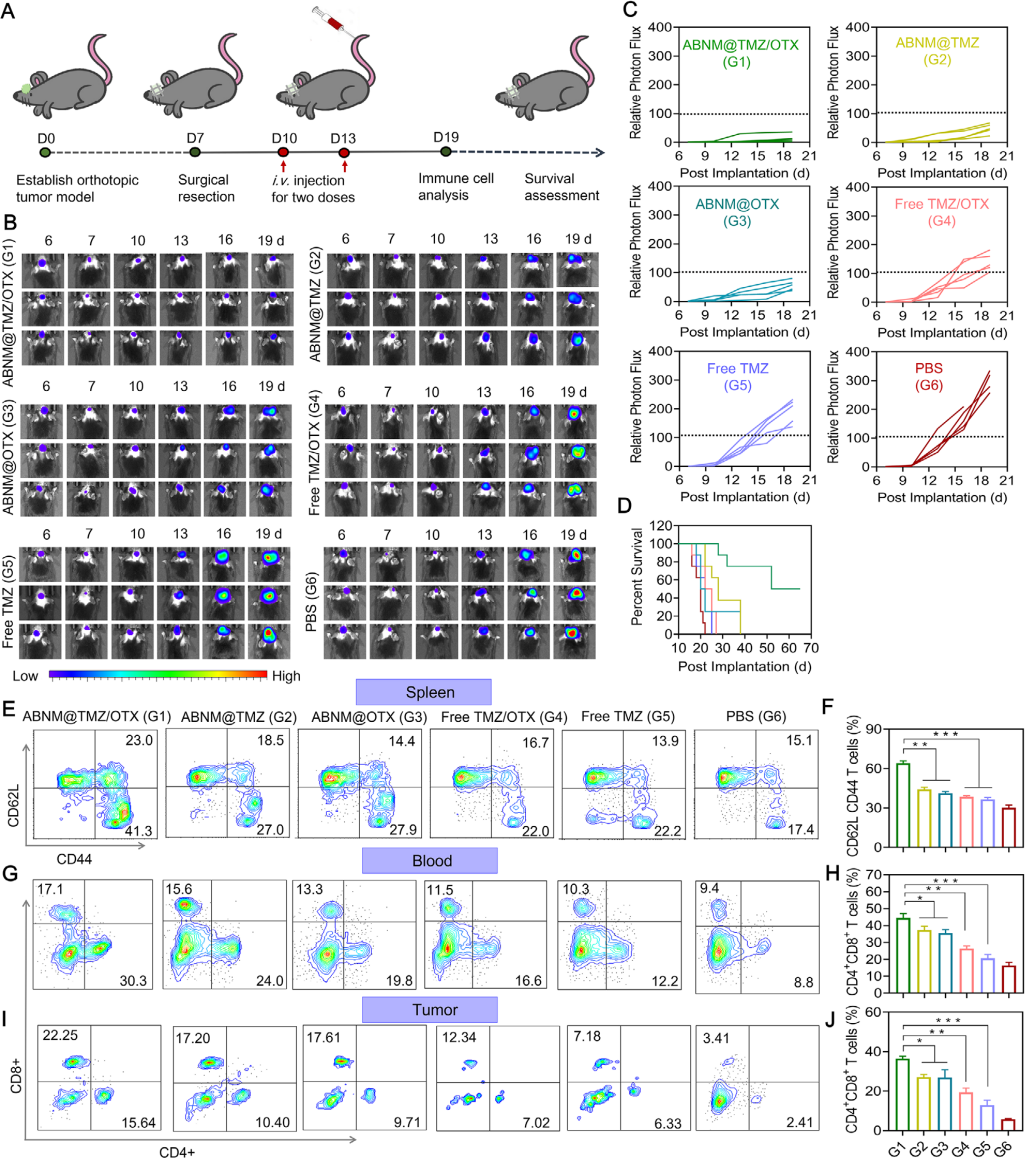
Effective chemo‐immunotherapy in tumor‐recurrence GL261 model mice. (A) Schematic timeline of the GL261‐Luc GBM anti‐recurrence efficacy study. Tumors were initially surgically resected on day 7 and mice were then intravenously injected with ABNM@TMZ/OTX, ABNM@TMZ, ABNM@OTX, free TMZ/OTX, free TMZ (5.0 mg TMZ equiv./kg and/or 5.0 mg OTX equiv./kg) or PBS on days 10 and 13 post tumor implantation. (B) Bioluminescent signal images of mice bearing orthotopic GL261‐Luc GBM recurrence tumor after treatment as described in (A). (C) Quantified luminescence levels of orthotopic GL261‐Luc tumor in mice following treatment as described in (A). (D) Mice survival rates. (E) Representative flow cytometry dot plots and (F) statistical data of the expression of memory immune cells in the spleen from GL261‐Luc (gated on CD62L and CD44 T cells). Quantification of the infiltration of CD4^+^ and CD8^+^ T cells in (G,H) blood and (I,J) tumor by flow cytometry in recurrent GL261‐Luc model mice on day 19 after treatment as described in (A). Expression of CD4^+^ and CD8^+^ markers by staining with anti‐CD4‐FITC and anti‐CD8‐APC in tumor and blood. Mice treated with PBS were used as controls

As GBM tumor recurrence is a major clinical issue leading to poor prognosis,^[^ ^25^ ^]^ we further assessed the combinational chemoimmunotherapy effects of the biomimetic nanomedicines ABNM@TMZ/OTX in a recurrence tumor model (Figure 5A). Tumors were surgically resected, and mice were subsequently treated with two doses of ABNM@TMZ/OTX or other treatments. Compared to mice treated with free drugs (free TMZ, or free TMZ/OTX), mono‐drug loaded nanomedicines, ABNM@TMZ and ABNM@OTX, could partly delay recurrence of tumor proliferation. However, ABNM@TMZ/OTX showed impressive therapeutic effects (Figure 5B,C). The median survival time of mice treated with ABNM@TMZ/OTX was prolonged to 58.5 days, which was significant improved than mice receiving ABNM@TMZ (28 days), ABNM@OTX (21 days), free TMZ/OTX (23.5 days), and free TMZ (23.5 days) or PBS (20 days), respectively (Figure 5D).

T cell immune responses were then investigated on day 19 after two doses of nanomedicine treatment. Notably, numbers of CD62L CD44 memory T cells were increased approximately 1.5‐fold in the spleens of mice receiving ABNM@TMZ/OTX compared with spleens from mice receiving mono‐drug formulations (Figure 5E,F). ABNM@TMZ/OTX also increased both the percentage and absolute numbers of activated CD8^+^ and CD4^+^ T cells in blood and tumor (Figure 5G–J), confirming that prevention of GBM re‐occurrence was achieved by activating anti‐tumor immune responses together with TMZ chemotherapy.

### 2.10 Biosafety evaluation of ABNM@TMZ/OTX

**FIGURE 6:**
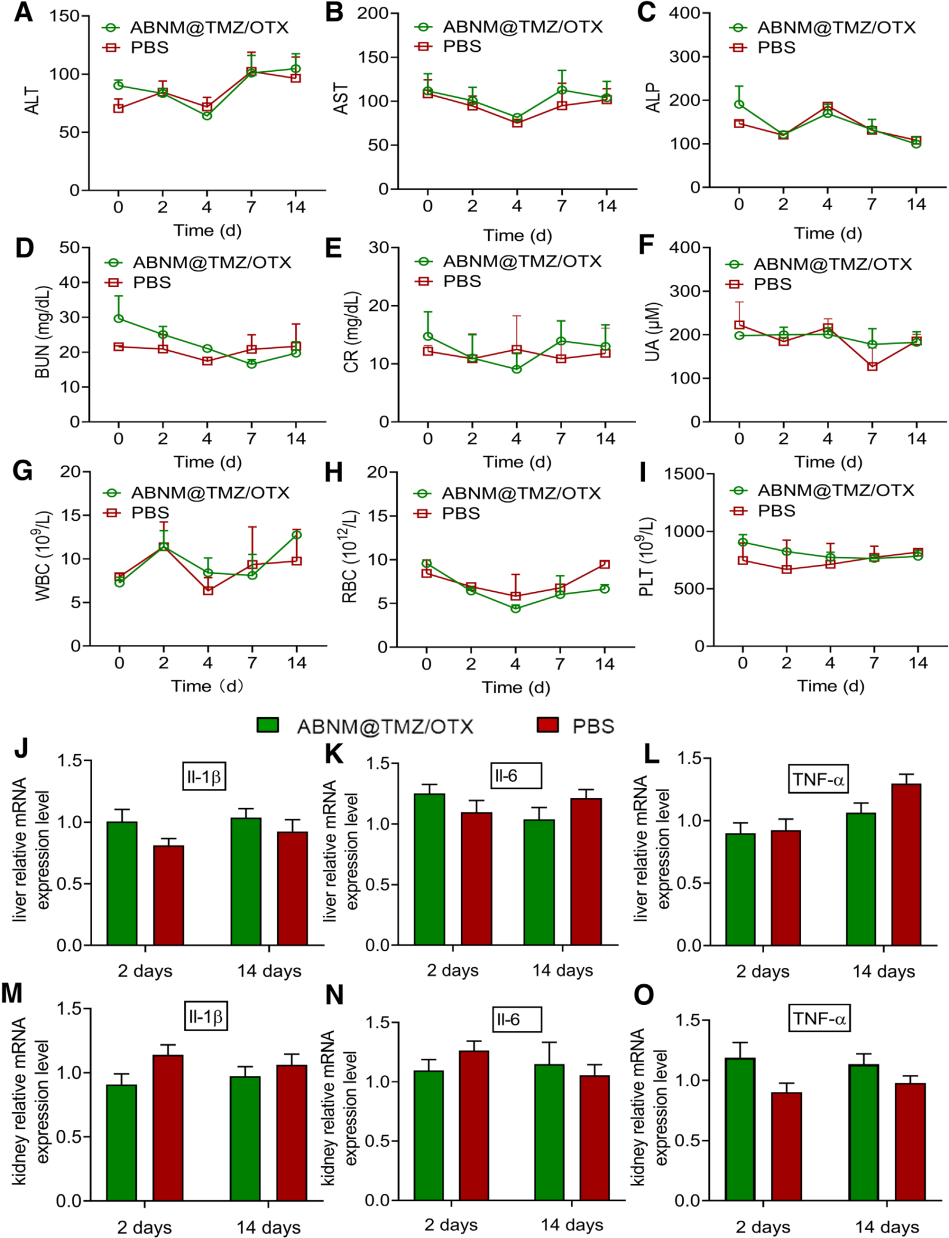
Biocompatibility evaluation of ABNM@TMZ/OTX. (A) Examination of plasma ALT, (B) AST, (C) ALP, (D) BUN, (E) CR, (F) UA contents after receiving ABNM@TMZ/OTX via tail vein (5 mg TMZ equiv./kg, 5 mg OTX equiv./kg). Routine blood examinations include (G) WBC counts, (H) RBC counts, and (I) PLT counts. Levels of pro‐inflammatory cytokines Il‐1β, Il‐6, and TNF‐α in liver (J–L) and kidney (M–O) were quantified after receiving ABNM@TMZ/OTX or PBS at days 2 and 14

The biocompatibility of ABNM@TMZ/OTX was assessed by routine blood and biochemistry (Figure 6A–I). To further evaluate induction of inflammatory processes of potential concern by nanomedicine treatment, pro‐inflammatory cytokines (Il‐1β, Il‐6, and TNF‐α) were analyzed in liver and kidney (Figure 6J–O). The results showed no significant difference between ABNM@TMZ/OTX and PBS groups, reflecting the good biocompatibility of these nanomedicines.

## 3 DISCUSSIONS

GBM is the most lethal and common malignancy that occurs in the central nervous system and currently, lacks effective and safe treatments.^[^ ^26^ ^]^ The present standard treatment of primary GBM features a median overall survival of only 14.6 months, highlighting the need to further improve GBM therapeutic outcome.^[^ ^27^ ^]^ As the only FDA‐approved chemodrug used clinically for the treatment of newly diagnosed GBM,^[^ ^4^, ^28^ ^]^ TMZ nonetheless suffers from short plasma circulation time, unstable physiochemical properties, and lack of GBM targeting capability.^[^ ^29^ ^]^ The additional key problem of tumor resistance reflects its mechanism of action. TMZ is an alkylating agent which binds to the DNA in tumor cells and interferes with tumor cell division and growth. The development of drug resistance markedly compromises the therapeutic efficacy of TMZ.^[^ ^25^, ^30^ ^]^

Due to the complex etiopathogenesis of GBM, combinational therapeutic approaches targeting more than one pathogenetic pathway should be more effective for the clinical management of GBM. Given the current irreplaceability of TMZ and its unavoidable drug resistance, we sought a therapeutic partner which could overcome TMZ drug resistance and ideally bring therapy from other dimensions to synergistically enhance the GBM therapeutic efficacy. Accordingly, OTX was selected for GBM synergistic treatment for three main reasons: 1) it is able to turn “cold” immunosuppressive GBM into T cell inflamed “hot” tumor via inhibiting the PD‐1/PD‐L1 pathway, leading to powerful immunotherapy; 2) OTX can inhibit the DNA repairment via acting as an inhibitor of bromodomain protein 4 (BRD4), thus reducing development of TMZ drug resistance; 3) OTX itself also has innate antitumor activity.

To facilitate brain‐targeted co‐delivery and GBM targeting of TMZ and OTX, we co‐loaded TMZ and OTX with pH sensitive biocompatible polymer and then camouflaged the TMZ/OTX bearing core with red blood cell membrane (RBCm) to prevent immunogenicity and yield the biomimetic ABNM@TMZ/OTX nanomedicine. As expected, effective synergetic GBM treatment was achieved by ABNM@TMZ/OTX, which showed a low Chou‐Talalay combination index in GL261 cell proliferation studies (Figure 1G). The next key design element involved a “two birds, one stone” strategy in which the outer shell of ABNM@TMZ/OTX was functionalized with ApoE peptide which specifically binds to LDLR and LRP‐1 receptors that are highly expressed on both the endothelial cells of the BBB and GBM cells, endowing ABNM@TMZ/OTX nanomedicine with both high BBB penetration and GBM targeting capability. This greatly enhances the ability of OTX to trigger immunotherapy by facilitating binding to PD‐L1 on the surface of GBM cells. Due to the excellent BBB penetration and OTX accumulation in GBM (7.6% of injected dose (ID), Figure 3I), the level of dendritic cells in lymph node and anti‐tumor T cells (CD4^+^ and CD8^+^) in blood and tumor microenvironment were significantly increased (Figure 2), leading to significantly synergistic GBM treatment in GL261 GBM bearing mice.

To better evaluate the synergistic treatment effect of ABNM@TMZ/OTX, we established a GBM‐recurrence model that better recapitulates the clinical therapy of GBM. Interestingly, our biomimetic ABNM@TMZ/OTX showed much better synergistic treatment (Figure 5) than that achieved in the primary GL261 GBM model, indicating the promising clinical application of ABNM@TMZ/OTX. Accordingly, co‐delivering TMZ with OTX in brain and targeting GBM leads enhanced GBM synergistic inhibition in three different ways: 1) Co‐delivery of OTX into brain with the developed smart brain delivery system inhibits DNA repair to restore GBM sensitivity to TMZ in a safe and effective way, resulting in significantly synergetic GBM inhibition (Figure 1D); 2) TMZ can also induce activated anti‐tumor immune responses via induction of ICD in GBM therapy,^[^ ^31^ ^]^ which is often weak with “cold” GBM mainly attributable to immunosuppressive microenvironment (Figure 2B–G, Figure 5E–J). We solved this issue via co‐delivering epigenetic bromodomain inhibitor OTX which suppresses the expression of PD‐L1 and generates robust anti‐tumor responses, turning the “cold” immunosuppressive GBM into T cell inflamed “hot” tumors with significant increased dendritic cells (CD80, CD86), anti‐tumor T cells (CD4^+^, CD8^+^), and results in the second synergetic inhibition effect for GBM treatment (Figure 2A): 3) OTX itself can also induce GBM cell apoptosis via inducing cell cycle arrest which improves the GBM therapy of TMZ. As compared with commercial PD‐L1 anti‐bodies, OTX as the small molecule epigenetic bromodomain inhibitor that could not only inhibit the interaction of PD‐1 and PD‐L1, but also suppress tumor cell proliferation, providing a potent alternative for immune checkpoint inhibitors. Collectively, brain co‐delivery of TMZ with OTX can trigger three different syngenetic mechanisms of GBM inhibition, resulting in significantly extended median survival time in both orthotopic primary GBM (44 days vs. 20 days) and recurrent GBM (58.5 days vs. 20 days) models with negligible body weight loss. Another important feature of ABNM@TMZ/OTX was the demonstration of safety in vivo.

In conclusion, we developed biomimetic ABNM@TMZ/OTX as a non‐invasive brain targeting nanomedicine for effective and safe synergistic GBM treatment. The successful development of ABNM@TMZ/OTX solves brain‐drug delivery problems currently limiting clinical efficacy and confirms that combined chemotherapy and immunotherapy are more effective cancer therapy than single therapies in both primary and recurrence models of orthotopic GBM. Our biomimetic nanomedicines provide a new multifunctional platform to treat chemo‐drug and immune resistant cancers including GBM.

## 4 EXPERIMENTAL SECTION

Experimental details are provided in the Supporting Information.

## CONFLICT OF INTEREST

Bingyang Shi is a member of the *Exploration* editorial board. All authors declare no conflict of interest.

## Supporting information

Supporting Information
