# Supplementary material for: Brain co‐delivery of first‐line chemotherapy drug and epigenetic bromodomain inhibitor for multidimensional enhanced synergistic glioblastoma therapy
Source: Exploration (Beijing). 2022 Apr 19;2(4):20210274. doi: 10.1002/EXP.20210274 (PMC10190947; doi:10.1002/EXP.20210274)
Supplement: Supplementary file 1 — Supporting Information [file EXP2-2-20210274-s001.docx]

**Supplementary Materials for**

**Brain Co-delivery of First-line Chemotherapy Drug and Epigenetic Bromodomain Inhibitor for Multidimensional Enhanced Synergistic Glioblastoma Therapy**

Yanjie Liu^1^, Wendie Wang^1^, Dongya Zhang^1^, Yajing Sun^1^, Fangzhou Li^2^, Meng Zheng^1^, David B. Lovejoy^3^, Yan Zou^1,3*^, and Bingyang Shi^1,3*^

^1^Henan-Macquarie Uni Joint Centre for Biomedical Innovation, Academy for Advanced Interdisciplinary Studies, Henan Key Laboratory of Brain Targeted Bio-nanomedicine, School of Life Sciences, Henan University, Kaifeng, Henan, 475004, China.

^2^CAS Key Laboratory for Biomedical Effects of Nanomaterials and Nanosafety, CAS Center for Excellence in Nanoscience, National Center for Nanoscience and Technology of China, Beijing, 100190, China

^3^Centre for Motor Neuron Disease Research, Macquarie Medical School, Faculty of Medicine, Health and Human Sciences, Macquarie University, Sydney, NSW 2109, Australia.

*Correspondence: yzou@henu.edu.cn (Y. Zou), bingyang.shi@mq.edu.au (B. Shi).

**Materials and methods**All chemicals were purchased from Sigma-Aldrich unless otherwise noted. Lipid-tethered polyethylene glycol (DSPE-PEG, 2k) and lipid-tethered polyethylene glycol-maleimide (DSPE-PEG-Mal, 2k) were synthesized by Jenkem Technology (Beijing, China). Apolipoprotein E peptide [ApoE-SH, (LRKLRKRLL)_2_C, 95%] was obtained from China peptide Co., Ltd. (Suzhou, China). OTX015 was purchased from APExBIO (Houston, USA), Penicillin streptomycin, DMEM, fetal bovine serum (FBS) and 0.25% (w/v) trypsin solution were purchased from Gibco BRL (Gaithersberg, MD, USA). Triton X-100 was provided by Beyotime Biotechnology Co., Ltd. (Nantong, China). 1, 1’-dioctadecyl-3, 3, 3’, 3’-tetramethylindotricarbocyanine iodide (DiR) was the product of Biotium (Invitrogen, USA). UltraPure water was rendered basic (~ pH 8) by the addition of NaOH solution. PD-L1 PE-conjugated antibody (Thermo Fisher Scientific, ab210189), IgG1 PE-conjugated antibody (Thermo Fisher Scientific, ab91357), γH2AX antibody (EMD Millipore, Catalog No. 05-636), anti-CRT antibody (Cell Signaling Technology, 62304S), anti-HMGB 1 (Thermo Fisher Scientific, ab79823), anti-CD11c-FITC (eBioscience, 11-0114-85), antiCD80-PE (eBioscience, Clone: 16-10A1, Catalog: 12-0801-85) and anti-CD86-APC (eBioscience, Clone: GL1, Catalog: 17-0862). anti-CD3-PerCP-Cy5.5 (eBioscience, Clone: 145-2C11, Catalog: 45-0031), anti-CD4-FITC (eBioscience, Clone: GK1.5, Catalog: 11-0041) and anti-CD8-PE (eBioscience, Clone: 53-6.7, Catalog: 12-0081), FITC anti-mouse CD3 antibody (Biolegend, 100306), PE anti-mouse/human CD44 antibody (Biolegend, 103008), APC anti-mouse CD62L antibody (Biolegend, 104412), PerCP-Cy5.5 anti-mouse CD8a antibody (Biolegend, 100734), Anti-LDLRs antibody (Abcam, ab52818), Anti-LRP1 antibody (Abcam, ab92544).

**Preparation of RBC membrane (RBCm) and ligand-inserted RBCm membrane (ApoE-RBCm)**. Red blood cell membrane and ligand modified red blood cell membrane were prepared in accordance with our previous report. (*1, 2*) To obtain ApoE functionalized RBCm (ApoE-RBCm), the pellets (100 μL) were incubated with ApoE-PEG-DSPE (50 µg) for 30 min. ApoE-PEG-DSPE was prepared by dissolving Mal-PEG-DSPE and ApoE in PBS buffer (pH 7.4) at a molar ratio of 1:3 followed by overnight reaction at 37℃. The degree of Ang conjugation was determined to be 90% by Micro BCA protein assay kit (Thermo scientific).

**Fabrication of ABNM@TMZ/OTX**. Firstly, naked nanoparticles were loaded with TMZ and OTX (NM@TMZ/OTX), and prepared by self-assembly. The synthesis of acetal-dextran and FITC labeled acetal-dextran were the same as our previous report.(*2*) Briefly, tetrahydrofuran (THF) solution (200 μL) containing acetal-dextran (1 mg) was mixed with a dimethylsulfoxide (DMSO) solution (22 μL) containing TMZ (250 μg) and OTX (136 μg) to yield a theoretical drug loading content (DLC) of 20 *wt.*% and 12 *wt.*%, respectively. The mixture was then dropwise added to ddH_2_O (1 mL, pH = 8). The solution was stirred at room temperature for 3 h to evaporate the solvent. Free drugs were washed away by dialysis for 12 h. The drug loading efficiency (DLC) of TMZ was analyzed by high performance liquid chromatography (HPLC, Agilent technology, 1260 Infinity II) using a mixture of methanol/0.5% acetic acid (v/v) as eluent (v/v = 10/90, flow rate: 1 mL/min). The OTX was quantified similarly by HPLC using the acetonitrile and water as eluent (v/v = 50/50, flow rate: 1 mL/min), the UV absorbance of TMZ and OTX was 329 nm and 254 nm, respectively.

To encapsulate NM@TMZ/OTX into ApoE functionalized RBCm (ApoE-RBCm), vesicles harvested from 200 μL blood were mixed with 1 mg NM@TMZ/OTX in 1 mL of ddH_2_O as previously reported calculation.(*2*) The pH value was adjusted to 7.4 by addition of NaOH, subjected to sonication at a frequency of 42 kHz and 100 W power for 2 min. Free DSPE-PEG-ApoE was removed by dialysis. The size and zeta potential of nanomedicines were determined at 25 °C using dynamic light scattering (DLS; Zetasizer Nano-ZS, Malvern Instruments). For transmission electron microscopy (TEM) imaging, 10 µL of the nanomedicine solution was deposited onto a glow-discharged carbon-coated grid. After ten minutes the grid was washed with 10 drops of distilled water, a drop of 1% uranyl acetate stain was added to the grid. The grid was subsequently dried and visualized using TEM (JEM-2010HT, Japan).

**Receptor expression on GBM cells.** The expression levels of low-density lipoprotein receptor-1 (LRP1) and low-density lipoprotein receptor family (LDLRs) in GL261 GBM cells and normal HA1800 astrocytes were measured by western blotting assays in accordance with literature methods.(*3*) Polyvinylidene difluoride membranes were blocked with 5% bovine serum albumin (BSA) at room temperature (1 h) and then incubated at 4 °C with primary antibodies (rabbit anti-human LRP1 or LDLR; 1:1000 dilution) overnight, followed by incubation with secondary antibody (phycoerythrin-labeled goat antirabbit IgG; 1:10,000 dilution). Fluorescence signals were measured using Super Signal ECL on a Bio-Rad ChemiDoc MP System. β-actin antibody (1:1000 dilution) was used as loading control.

**Drug release.** The *in vitro* release of TMZ or OTX from ABNM@TMZ/OTX nanomedicines was studied using a dialysis tube (MWCO 12,000) with shaking (200 rpm) at 37 °C under three different conditions, (i) phosphate buffered saline (PBS, 10 mM, pH 7.4), (ii) acetate buffer (10 mM, pH 6.5), (iii) acetate buffer (10 mM, pH 5.0) (mimicking the intracellular acidic trafficking pathway). ABNM@TMZ/OTX was dialyzed with 25 mL of release media. At each time point, the solution outside the dialysis membrane (5 mL) was withdrawn and replaced with the same volume of fresh media. The TMZ or OTX concentration in the solution was determined by HPLC.

**Cell uptake experiment**. GL261 cells were seeded in a 6-well plate (1× 10^6^ cells/well) and incubated with FITC labelled ABNM, BNM, or NM at 37 °C for 4 h. The cells were digested by 0.25% (w/v) trypsin and 0.03% (w/v) EDTA. The suspensions were centrifuged at 1000 ×g for 3 min, washed twice with PBS, and then re-suspended in 500 μL of PBS. Fluorescence histograms were immediately recorded with a flow cytometer (CytoFlex, Beckman) and analyzed using Cell Quest software based on 10,000 gated events. The gate was arbitrarily set for the detection of FITC fluorescence.

**Intracellular release.** The intracellular release behaviour of FITC labelled ABNM were tracked with confocal laser scanning microscopy (CLSM) using GL261 cells. The cells were seeded on microscope slides in a 24-well plate (5 × 10^5^ cells/well) using DMEM medium supplemented with 10% FBS, 1% glutamine, antibiotics penicillin (100 IU/mL), and streptomycin (100 mg/mL). The cells were incubated with FITC labelled ABNM, BNM, or NM at 37°C for 4 h. Then the culture medium was removed, the cells on microscope plates were washed with PBS for three times, fixed with 4% paraformaldehyde solution for 15 min and stained with 4,6-diamidino-2-phenylindole (DAPI) for 10 min and finally mounted with glycerol. The cell uptake of ABNM in bEnd3 endothelial cells was similar as described above. The fluorescence images were obtained using a confocal microscope (Zeiss 880).

***In vitro* BBB penetration.** The *in vitro* BBB model was established by seeding bEnd3 cells (5 × 10^4^/well) on the upper chamber of culture inserts (Corning, NY, USA) which were put into the 24-well plates. Then Cy5 labeled ABNM and BNM (Cy5: 5 μg/mL) were added to the upper chamber, and the FBS free medium was added to the lower chamber. At determined intervals (1, 2, 4, 8, 12 and 24 h), the Cy5 fluorescence in the lower chamber were analyzed using a microplate reader. The cumulative transport ratio was calculated according to the initial feeding amount of Cy5 labeled nanomedicines.

**OTX and TMZ sensitivity assays.** For immunofluorescence of γH2AX DNA damage foci, GL261 cells were seeded on glass coverslips. Cells were exposed to ABNM@TMZ/OTX, ABNM@TMZ, ABNM@OTX, free TMZ, free OTX or free TMZ/OTX (150 μM TMZ, 400 nM OTX) for 72 h, then washed three times with PBS and fixed using 4% paraformaldehyde for 20 min. Cells were blocked with goat serum in Triton X-100 and PBS for 1 h and incubated in γH2AX antibody (EMD Millipore, Catalog No. 05-636) at 1:1000 dilution overnight at 4 °C. Coverslips were washed three times in PBS and incubated in secondary Alexafluor antibody (Invitrogen, Catalog No. A11001) and DAPI counterstain (Thermo Fisher, Catalog No. 62248) both at 1:1000 dilution for 1 h at room temperature. Coverslips were mounted onto glass slides using glycerol and images were taken using a confocal microscope.

**Verification of synergistic anti-GBM activity induced by OTX with TMZ by MTT cell proliferation assays.** For TMZ and OTX C.I. assays, cells were seeded onto 96-well plates in ABNM@TMZ/OTX, ABNM@TMZ, ABNM@OTX, free TMZ, free OTX or free TMZ/OTX for 72 h. The concentrations of TMZ ranging from 0 to 160 μM and OTX ranging from 0 to 1.6 μM. Cell viability was measured using MTT assay and C.I. values were calculated using the Chou-Talalay method with CompuSyn software.

**PD-L1 inhibition.** To investigate OTX-induced downregulation of PD-L1 on the membrane of GL261 cells, the cells were seeded in 6-well culture plates at a density of 3 × 10^4^ cells/well and cultured overnight. The cells were incubated with ABNM@TMZ/OTX, ABNM@TMZ, ABNM@OTX, free TMZ, free OTX, free TMZ/OTX (150 μM TMZ, 400 nM OTX) or PBS for 72 h. After that, the cells were digested by 0.05% trypsin and washed in PBS for 3 times. PD-L1 PE-conjugated antibody or IgG1 PE-conjugated antibody were added to the cell suspension solution on ice for 30 min, then the cells were washed with FACS buffer (PBS contained 0.5% BSA) for 3 times and examined using flow cytometry (CytoFlex, Beckman).

**Determination of immunogenic cell death (ICD) *in vitro*.** Calreticulin (CRT) expression, nuclear high mobility group protein 1 (HMGB 1) efflux and ATP release were assessed to verify the ICD effect of ABNM@TMZ/OTX on GL261 cells. To visualize CRT expression, GL261 cells were seeded in 24 well plates (5 × 10^4^ cells/well) and cultured overnight. Cell media was removed and supplemented with fresh media containing ABNM@TMZ/OTX, ABNM@TMZ, ABNM@OTX, free TMZ, free OTX or free TMZ/OTX (150 μM TMZ, 400 nM OTX) for 72 h. Cells were then ﬁxed and washed three times with cold PBS, and then stained with an anti-CRT antibody (cell signaling technology, 62304S) for 30 min at 4°C. Subsequently, the cells were further washed twice with PBS and incubated with Alexa Fluor 488-conjugated secondary antibody (Life technologies, USA) for 1 h. After staining with DAPI, confocal imaging was performed to visualize the CRT expression.

To examine HMGB 1 release, GL261 cells were incubated in 24 well plates (5 × 10^4^ cells/well) for 24 h. Cell culture media was removed and supplemented with fresh medium containing ABNM@TMZ/OTX, ABNM@TMZ, ABNM@OTX, free TMZ, free OTX or free TMZ/OTX (150 μM TMZ, 400 nM OTX) for 72 h. Cells were then washed with PBS for 3 times, fixed in 4% paraformaldehyde for 20 min and permeabilized with 0.1% Triton X-100 for 10 min. After that, the cells were incubated with primary antibody anti-HMGB 1 (ab79823) for 30 min and washed three times with cold PBS. The cells were then incubated with Alexa488-conjugated monoclonal secondary antibody for 1 h. Finally, the cells were stained with DAPI and examined by CLSM.

Extracellularly released ATP was quantified using the Chemiluminescence ATP Determination Kit. Briefly, GL261 cells were seeded into 24-well plates (5×10^4^ cells/well) and cultured for 12 h. Cell culture media was removed and supplemented with fresh medium containing ABNM@TMZ/OTX, ABNM@TMZ, ABNM@OTX, free TMZ, free OTX, or free TMZ/OTX (150 μM TMZ, 400 nM OTX). After 72 h incubation, the cell supernatant was collected and the released ATP was detected by the Chemiluminescence ATP Determination Kit, according to the manufacturer’s protocols.

**GL261-Luc orthotopic brain tumor model.** The luciferase-transfected mouse glioblastoma cell line GL261-Luc was kindly provided by Prof. Lei Zhang (Shanxi Normal University). Female C57BL/6 mice (six-week old, SiPeiFu (SPF) Beijing Biotechnology Co., Ltd) were anesthetized with 5% chloral hydrate and the surgical site was shaved and a total of 1 × 10^6^ tumor cells in 5 µL DMEM was stereotactically injected using a 10 µL Hamilton syringe into the left striatum over 1 min at the following coordinates: 0.5 mm anterior, 2 mm left lateral from bregma, and 3.5 mm deep. The wound was sealed by using surgical glue. The growth of the glioma was monitored by bioluminescence using an imaging system (IVIS, Lumina III; Caliper, MA, USA) after intraperitoneal injection of D-luciferin (75 mg/kg).

**ABNM@TMZ/OTX induced immune response *in vivo*.** Orthotopic GL261-Luc tumors were established in C57BL/6 mice as described above. When the bioluminescence intensity of mice tumor reached approximately 5×10^5^-1×10^6^ (p/sec/cm^2^/sr), the mice were randomly divided into two groups and intravenously injected with ABNM@TMZ/OTX or PBS. Serum samples were collected at predetermined time points. Serum concentrations of TNF-α, IFN-γ, and IL-6 were analyzed with enzyme linked immunosorbent assay (ELISA) kits (all from Neobioscience Co., Ltd., Shenzhen, China) according to the manufacturer’s protocols using samples collected at 24, 72, or 168 h.

To further investigate if ABNM@TMZ/OTX could accelerate DCs maturation *in vivo*, GL261 tumor bearing C57BL/6 (6-8 weeks) mice were injected with ABNM@TMZ/OTX, ABNM@TMZ, ABNM@OTX, free TMZ/OTX, free TMZ (5 mg TMZ equiv./kg, 5 mg OTX equiv./kg) or PBS via the tail vein. Three days post injection, mice were sacrificed, and the inguinal lymph nodes were collected for assessment by flow cytometry after co-staining with anti-CD11c-FITC, anti-CD80-PE and anti-CD86-APC antibodies according to manufacturer procedures.

To verify that ABNM@TMZ/OTX induced immune responses, mouse blood was collected 3 days post injection of nanomedicines. Blood was then centrifuged for 10 min at 1000 rpm to remove serum. Blood cells were incubated with red blood cell lysis buffer for 3 min at 4 °C and then centrifuged for 10 min at 1000 rpm to collect lymphocytes. The proportions of CD4^+^ or CD8^+^ T cells were examined using flow cytometry after staining with anti-CD3-PerCP-Cy5.5, anti-CD4-FITC and anti-CD8-PE antibodies according to the manufacturer procedures.

Tumors were also cut into small pieces and digested in RPMI-1640 medium including 2% FCS, collagenase (50 U/mL), HAse (100 μg/mL), and DNase (50 U/mL) for 2 h at 37 °C. Then, the digested suspensions were filtrated, centrifuged and washed with PBS (×3), then stained with anti-CD3-PerCP-Cy5.5, anti-CD4-FITC and anti-CD8-PE antibodies. Following PBS washing for three times, stained cells were suspended in PBS (0.5 mL) and measured by flow cytometry.

***In vivo* pharmacokinetics, imaging and tumor penetration.** To assess the blood circulation of ABNM@TMZ/OTX, we conducted pharmacokinetics studies in mice. Briefly, mice were randomly divided into three groups (n = 3 per group) and nanomedicines were injected through the tail vein (5 mg TMZ equiv./kg, 5 mg OTX equiv./kg). At different time points following injection, blood was collected and 100 µL lysis buffer (1% Triton X-100) was immediately added to the blood sample. Each blood sample was incubated with 0.5 mL of dimethyl sulfoxide (DMSO) solution at room temperature overnight. The mixture was then centrifuged at 12 krpm for 10 min and the TMZ and OTX content in the supernatant was determined by HPLC using a standard curve as described above. Data are presented as drug concentration per milliliter blood (μg/mL). Mice treated with 200 μL free TMZ/OTX solution were used as control.

In order to facilitate the fluorescence imaging of nanomedicines *in vivo*, 1,1′-dioctadecyltetramethyl indotricarbocyanine iodide (DiR) was loaded into nanomedicine by hydrophobic interaction, with a final dosage of 1.0 mg DiR equiv./kg. Mice with GL261 orthotopic xenografts were randomly grouped and injected with ABNM@DiR, BNM@DiR and NM@DiR. At predetermined time points (0, 1, 4, 8, 12, and 24 h) post *i.v.* injection, the mice were anesthetized with isoflurane and during the imaging acquisition process, 3% isoflurane anaesthesia was delivered via a nose cone system. Fluorescence images were acquired using a near-infrared fluorescence imaging system (IVIS III) at excitation of 747 nm and emission of 774 nm, and the images were analyzed using Lumia III software. For *ex vivo* imaging, mice were sacrificed at 8 h post-injection, and the major organs and brains were collected, and fluorescence images were acquired.

To evaluate the tumor penetration of the biomimetic nanomedicine, FITC labeled ABNM (1.0 mg FITC equiv./kg) was injected intravenously in C57BL/6 mice bearing GL261 orthotopic xenografts. At 8 h post injection, the brains were harvested, fixed in 4% formalin overnight, embedded in paraffin, and sectioned for immunofluorescent staining analysis. Sections were then counterstained with Alexa 647 tagged CD31 antibody, followed by washing three times with PBS and staining with DAPI (5 μg/mL) for 10 min. Finally, the stained sections were observed under a CLSM imaging system (Zeiss 880).

**Biodistribution of ABNM@TMZ/OTX.** To assess biodistribution, ABNM@TMZ/OTX was administrated intravenously via the tail vein (5 mg TMZ equiv./kg, 5 mg OTX equiv./kg) in C57BL/6 mice bearing GL261 orthotopic glioma at 15 days post-implantation. At 8 h post-injection, the tumor-bearing mice were sacrificed. The major organs including heart, liver, spleen, lung, kidney and brain tumor were collected, washed in PBS, and weighed. To quantify the amount of TMZ and OTX delivered to the tumor and different organs, the tumor block and organs were homogenized in 0.6 mL of 1% triton X-100 with a homogenizer at the frequency of 70k Hz for 6 min. Each tissue lysate was incubated with 0.9 mL DMSO at room temperature overnight. The samples were centrifuged at 10,000 rpm for 15 min and TMZ and OTX in the supernatant were quantified by HPLC and expressed as injected dose per gram of tissue (% ID/g).

***In vivo* therapy.** At day 12 after implantation. GL261 tumor bearing mice were randomly divided into six groups (n=11, 8 for survival assessment and 3 for histological analysis). ABNM@TMZ/OTX, ABNM@TMZ, ABNM@OTX, free TMZ/OTX, free TMZ (all at a dose equivalent of 5 mg TMZ equiv./kg and 5 mg OTX equiv./kg) or PBS were intravenous injected into the mice every other day. The tumor progression of the mice was tracked by the Lumina IVIS III system every other day via checking the luminescence of the brain tumor. The relative photon flux was normalized to the initial intensity, I/I_0_ (I_0_ is the tumor volume at day 12). On day 22, the treatment was terminated and three mice from each group were sacrificed. Tumors and major organs were collected and examined by Hematoxylin and eosin (H&E) staining. The tumors were harvested, fixed and sliced for Ki-67, Caspase 3, TUNEL, PD-L1, CD4^+^ and CD8^+^ staining. Signal intensity was quantified from >300 cells in tumors of mice per treatment condition using ImageJ. The survival and body weight of mice were individually measured.

**GBM surgical resection model.** To construct the surgical resection model, the tumors of the mice were resected at 7 d after tumor implantation assisted by the microscopy. Briefly, the tumor mass was identified under × 10 magnification and micro-surgically removed using a tissue microforce. Surgical tumor removal was continued until white walls, indicative of normal brain, were macroscopically apparent in the surgical cavity. Haemostasis was accomplished using gelfoam and prolonged irrigation. The operation time was approximately 20 min for each mouse. Mortality rate was lower than 5% and occurred within the first 2 d of surgery. Histological examination of the brain sections from the tumor-bearing mice two days after surgery showed that many islands of tumor cells persisted close to the surgical cavity. Body weights and survival time of the tumor-bearing mice were also monitored after surgery.

***In vivo* therapeutic efficacy of ABNM@TMZ/OTX for antitumor recurrence.** After tumor resection, the mice were weighed and randomly divided into six groups (n = 11, 8 for monitoring survival and 3 for analyzing immune cells). ABNM@TMZ/OTX, ABNM@TMZ, ABNM@OTX, free TMZ/OTX and free TMZ (dosage: 5 mg TMZ equiv./kg and 5 mg OTX equiv./kg), or PBS were intravenous injected into the mice on post implantation day 10 and day 13, optical imaging was performed using *in vivo* bioluminescent Lumina IVIS III systems. To study the mechanism, the immune cells in the recurrent tumor were analyzed on day 19. Tumors and blood were collected from each mouse, and single cell suspensions were obtained according to the method described above and then stained with anti-CD3-FITC, anti-CD4-PE, and anti-CD8-APC antibodies. Cells were sorted by a flow cytometer and analyzed with FlowJo software.

For the analysis of memory T cells, spleens were harvested from the survived mice on day 19 and stained with anti-CD3-FITC, anti-CD8-PerCP-Cy5.5, anti-CD62L-APC and anti-CD44-PE. Data analysis was carried out using FlowJo software and the gating method was illustrated in Figure S13.

**Blood routines and blood biochemistry.** Healthy Balb/C mice were weighed and randomly divided into two groups (n=3). ABNM@TMZ/OTX or PBS were intravenously injected via tail vein (dosage: 5 mg TMZ equiv./kg and 5 mg OTX equiv./kg). On day 2 and 14, mice were killed and the organs (liver and kidney) were collected for pro-inflammatory cytokines analysis. On day 0, 2, 4, 7, and 14, blood samples were taken from anesthetized mice via postcaval vein. The white blood cell (WBC), red blood cell (RBC), and platelets (PLT) were counted in each blood sample by using a pocH-100iV Diff hemocytometer (Sysmex). Moreover, the concentrations of alanine aminotransferase (ALT), aspartate aminotransferase (AST), alkaline phosphatase (ALP)，plasma urea (BUN), uric acid (UA), creatinine (CR) in plasma were quantified by Dri-Chem 7000IZ (Fuji Film Corp., Tokyo, Japan).

**Statistical analysis**

Results were analyzed by using GraphPad Prism software. Differences between two groups were assessed using unpaired t tests. For comparisons of three or more groups, differences among means will be analyzed using one-way analysis of variance (ANOVA) with the different treatments as the independent factor followed by Bonferroni (Dunn) post-hoc testing will test pair-wise comparisons between means. The level of statistical significance was set at *p* < 0.05. **p* < 0.05 was considered significant, and ***p*< 0.01 and ****p*< 0.001 were considered highly significant. All data were expressed as mean ± SD unless otherwise noted.

**Table S1.** Characterization of ABNM@TMZ/OTX in PB buffer.

| Nanomedicine | Size^a^  (nm) | PDI^a^ | ζ ^b^ (mv) | DLC  (*wt.*%) | | DLE  (%) | |
| --- | --- | --- | --- | --- | --- | --- | --- |
|  |  |  |  | TMZ^c^ | OTX^d^ | TMZ^c^ | OTX^d^ |
| ABNM@TMZ/OTX | 186 | 0.14 | -35 | 6.7 | 7.6 | 28.4 | 60.2 |
| BNM@TMZ/OTX | 182 | 0.08 | -25 |  |  |  |  |
| NM@TMZ/OTX | 168 | 0.16 | -38 | 6.9 | 7.8 | 29.6 | 62.2 |

Theoretical DLC of TMZ and OTX are 20 *wt.*% and 12 *wt.*%, respectively.

^a^Determined by DLS (10 mW He-Ne laser, 633 nm wavelength), ^b^ determined using Zetasizer Nano-ZS (Malvern instruments) at 25 °C in PB buffer (pH 7.4, 10 mM).

^c, d^Determined by HPLC, the UV absorbance of TMZ and OTX was 329 nm and 254 nm, respectively.


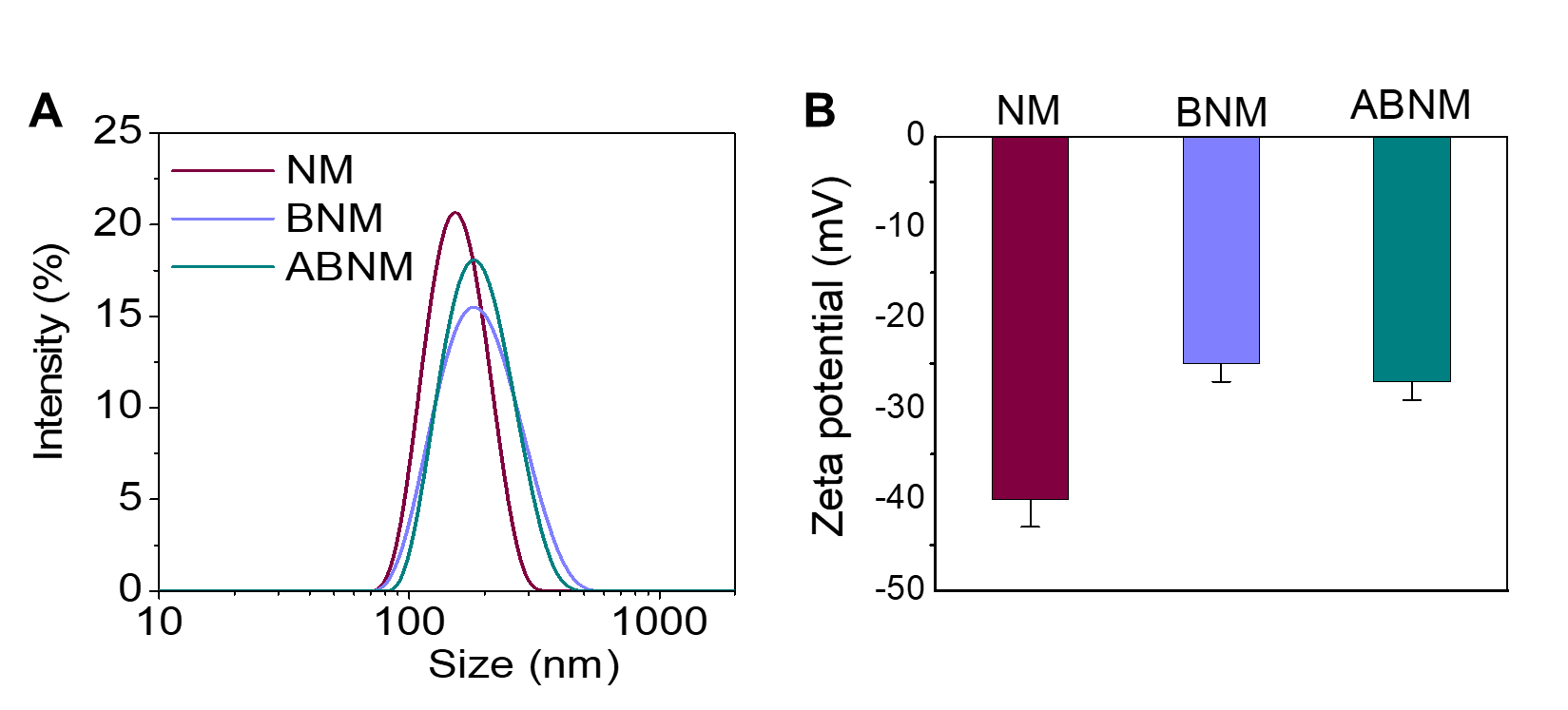


**Figure S1**. Size (**A**) and zeta potential (**B**) of NM, BNM and ABNM in PB buffer.


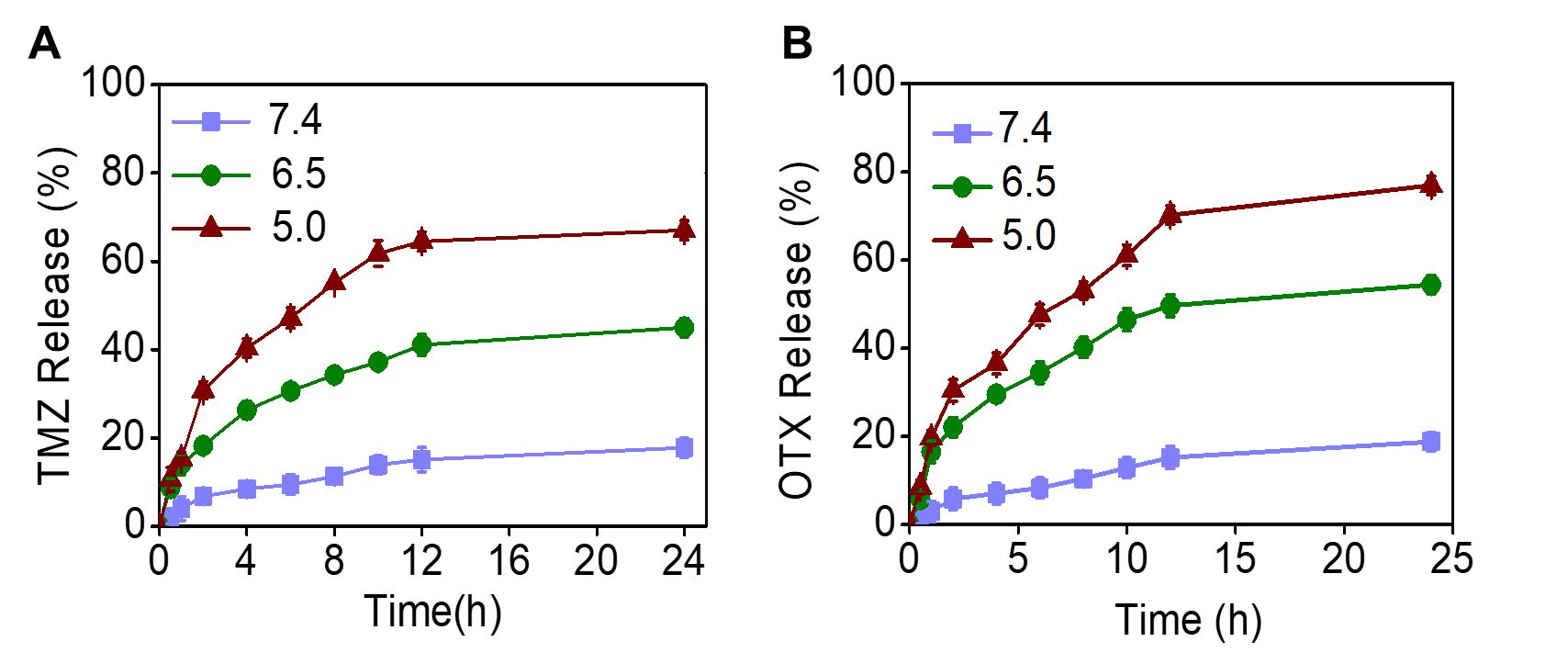


**Figure S2**. Cumulative release of TMZ (**A**) and OTX **(B)** from ABNM@TMZ/OTX in pH 5.0 and pH 6.5 acetic acid buffers at 37 °C or pH 7.4 phosphate buffer. Results are presented as mean ± standard deviation (n = 3).


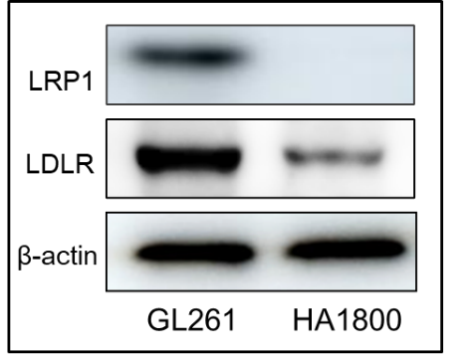


**Figure S3**. Evaluation of the expression of LDLRs (including LRP1 and LDLR) in GL261-Luc and HA1800 astrocytes determined by western blotting.


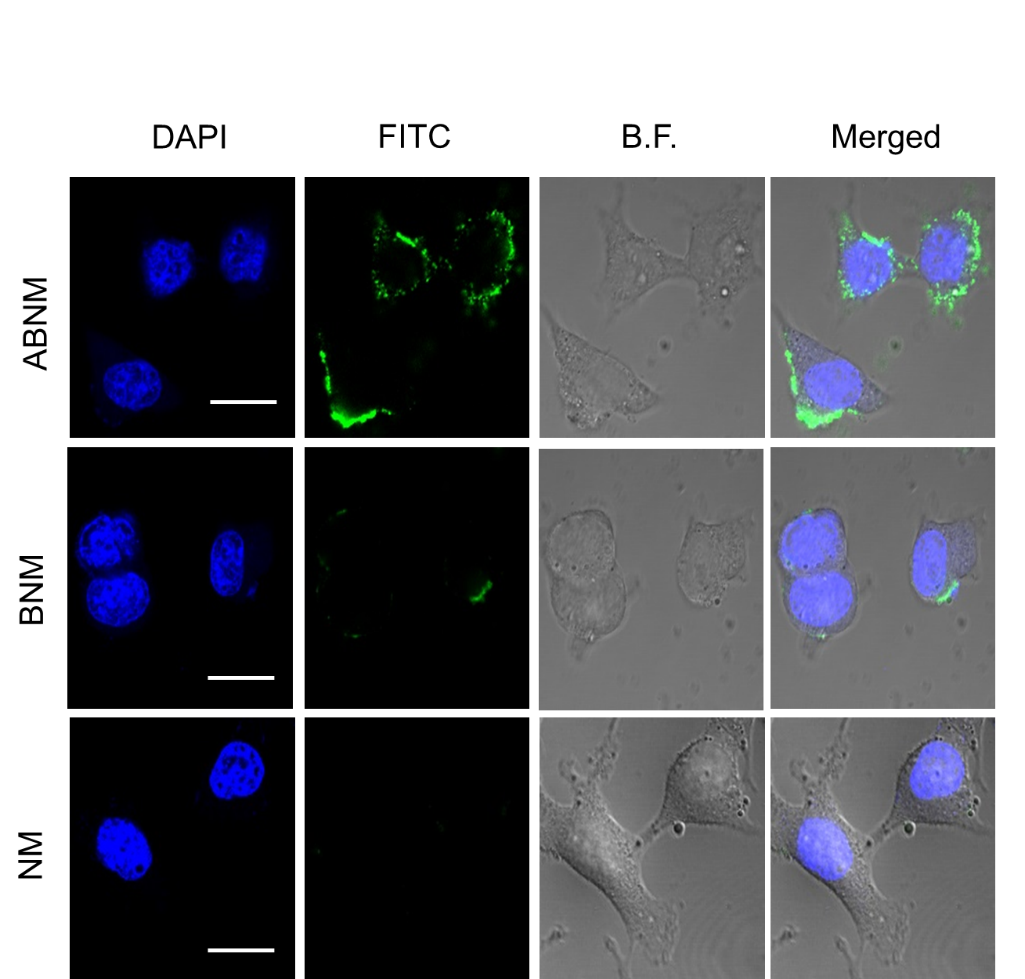


**Figure S4**. CLSM images of GL261 cells incubated with ABNM (FITC: 0.5 μg/mL) for 4 h. In each panel, images from left to right were cell nuclei stained by DAPI (blue), FITC (green), bright field (B.F.) and overlays of the three images. Scale bar: 10 μm.


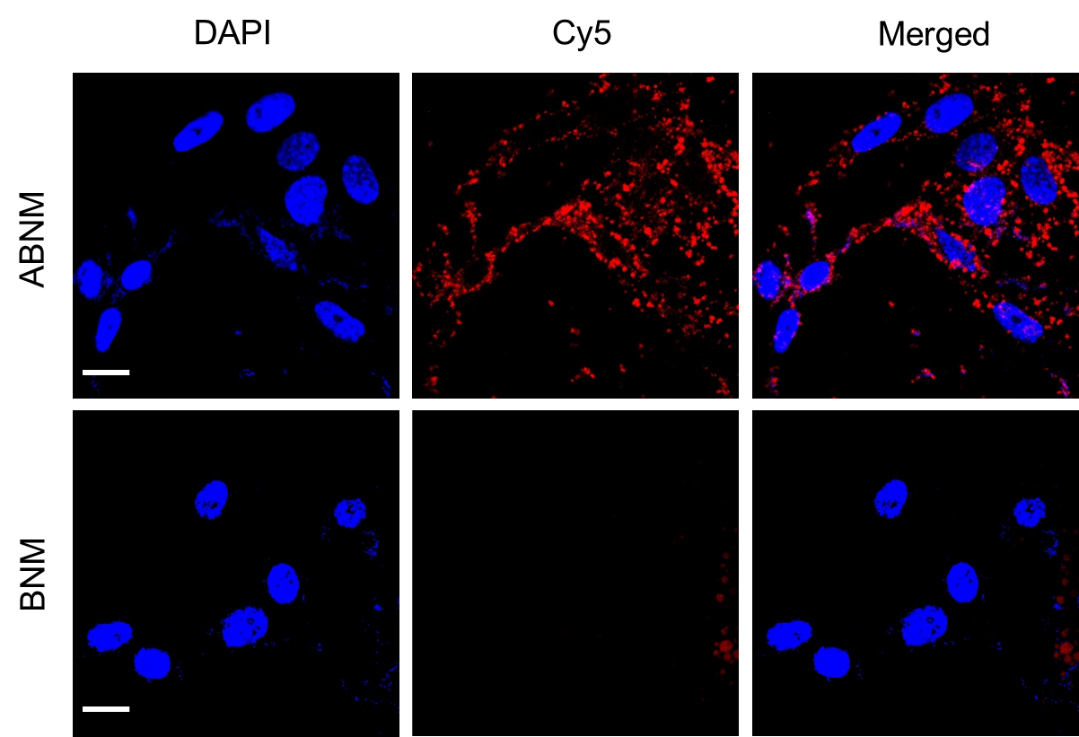


**Figure S5**. CLSM images of bEnd3 endothelial cells incubated with Cy5 labeled ABNM and BNM (Cy5: 5 μg/mL) for 4 h. Scale bar: 10 μm.

**Figure S6.** Cumulative BBB barrier transport ratio of Cy5 labeled ABNM and BNM (Cy5: 5 µg/mL). Data are presented as mean ± SD (n = 3, one-way ANOVA and Tukey multiple comparisons tests, **p* < 0.05, ***p* < 0.01, ****p* < 0.001).


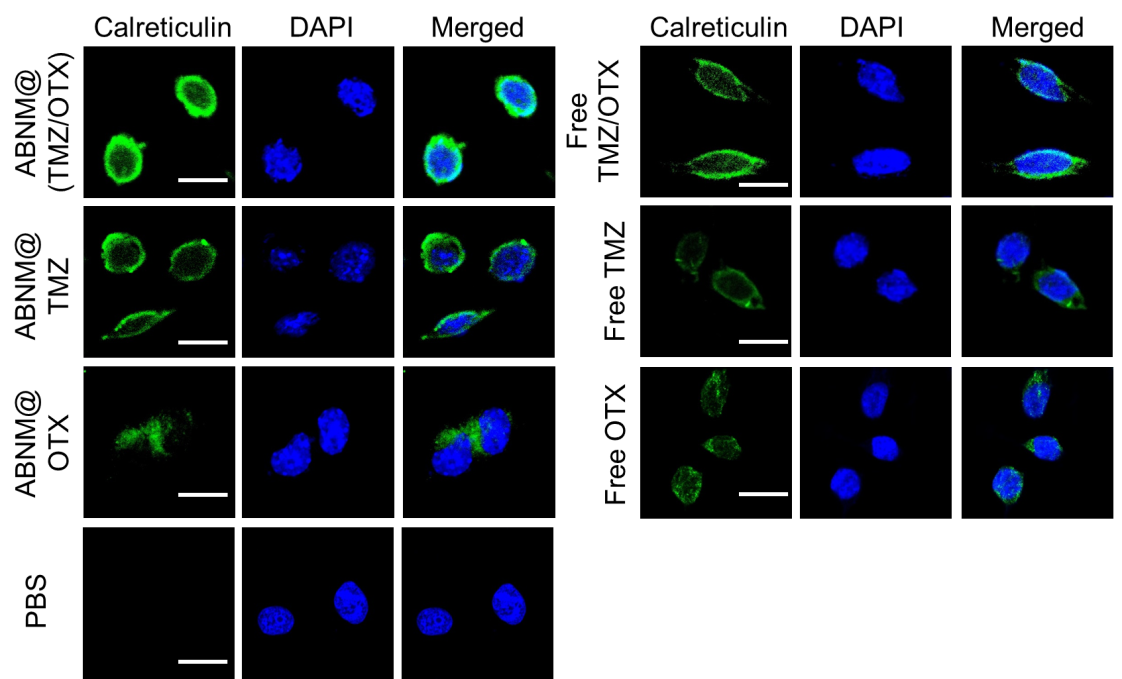


**Figure S7**. Calreticulin expression on the surface of GL261 cells treated with biomimetic nanomedicines ABNM@TMZ/OTX (150 μM TMZ, 400 nM OTX) for 72 h and observed by CLSM (scale bar: 10 µm).


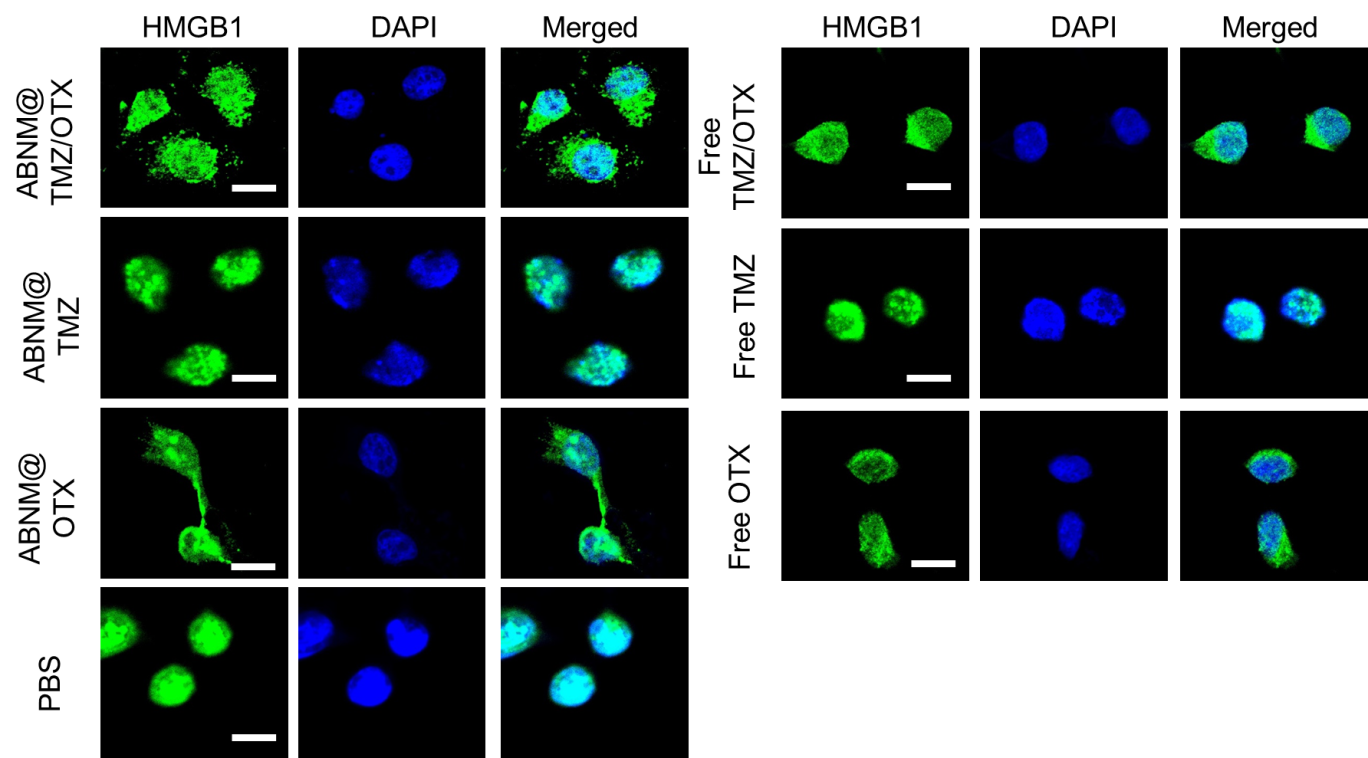


**Figure S8**. CLSM examination of ABNM@TMZ/OTX (150 μM TMZ, 400 nM OTX) induced nuclear HMGB1 efflux in the GL261 cells *in vitro* post 72 h incubation (scale bar: 10 µm).


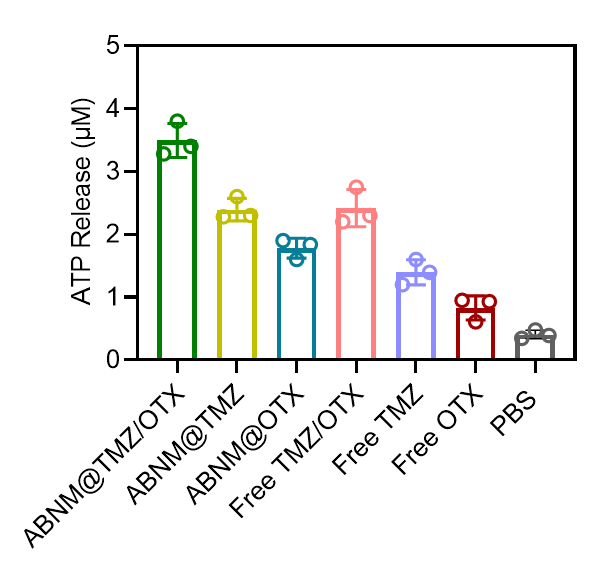


**Figure S9**. Induced release of ATP release by ABNM@TMZ/OTX (150 μM TMZ, 400 nM OTX) and control nanomedicines after 72 h incubation (n=3).


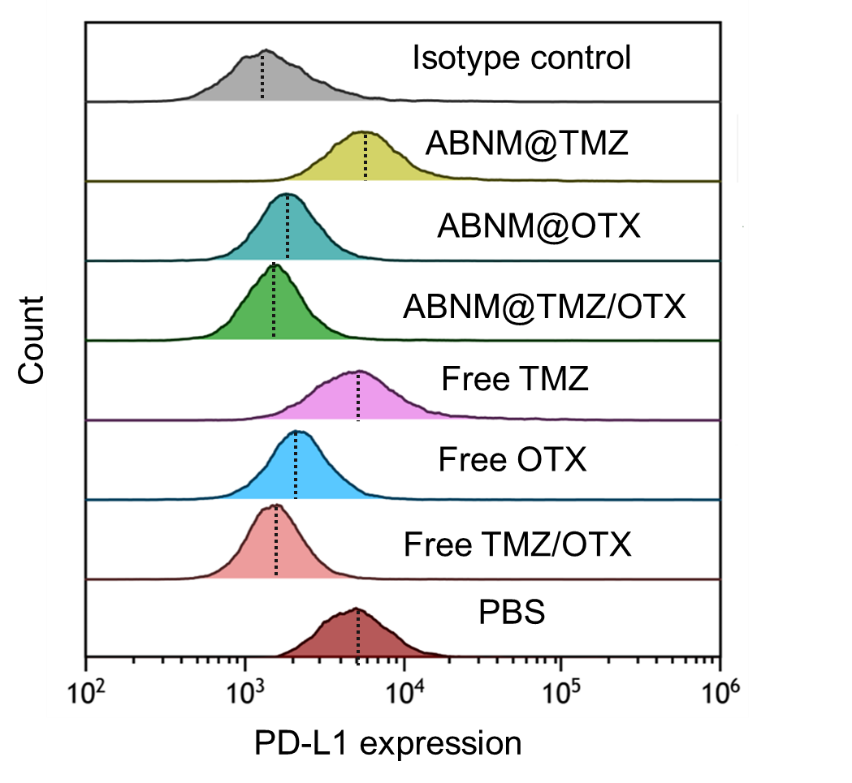


**Figure S10**. Flow cytometric analysis of ABNM@TMZ/OTX (TMZ: 150 μM, OTX: 400 nM) induced downregulation of PD-L1 on the membrane of GL261 cells after 72 h incubation.


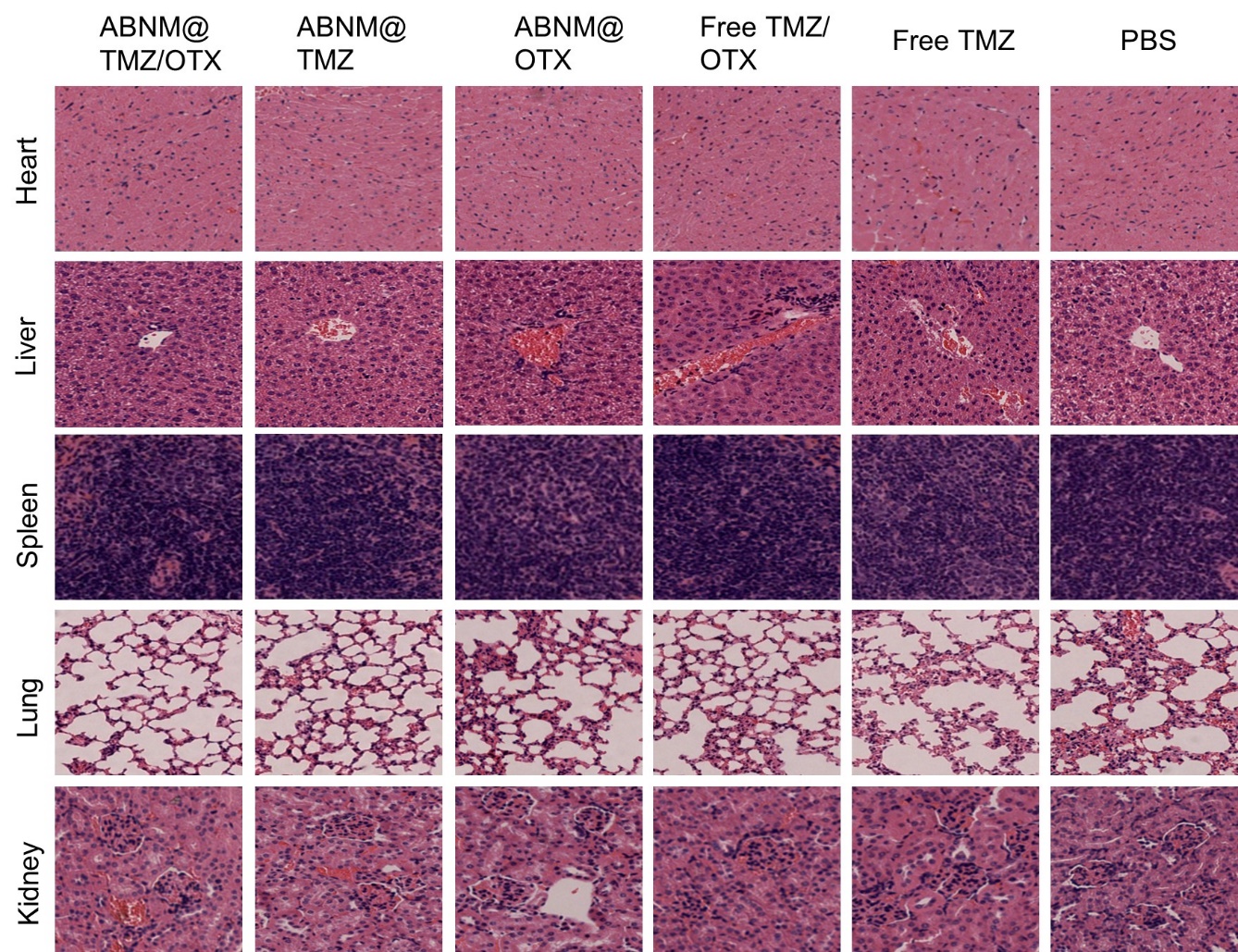


**Figure S11.** H&E staining of major tissues including heart, liver, spleen, lung and kidney taken from mice treated with ABNM@TMZ/OTX, ABNM@TMZ, ABNM@OTX, free TMZ/OTX, free TMZ or PBS. Mice were intravenously injected at a dose of 5.0 mg TMZ equiv./kg and/or 5.0 mg OTX equiv./kg via the tail vein.


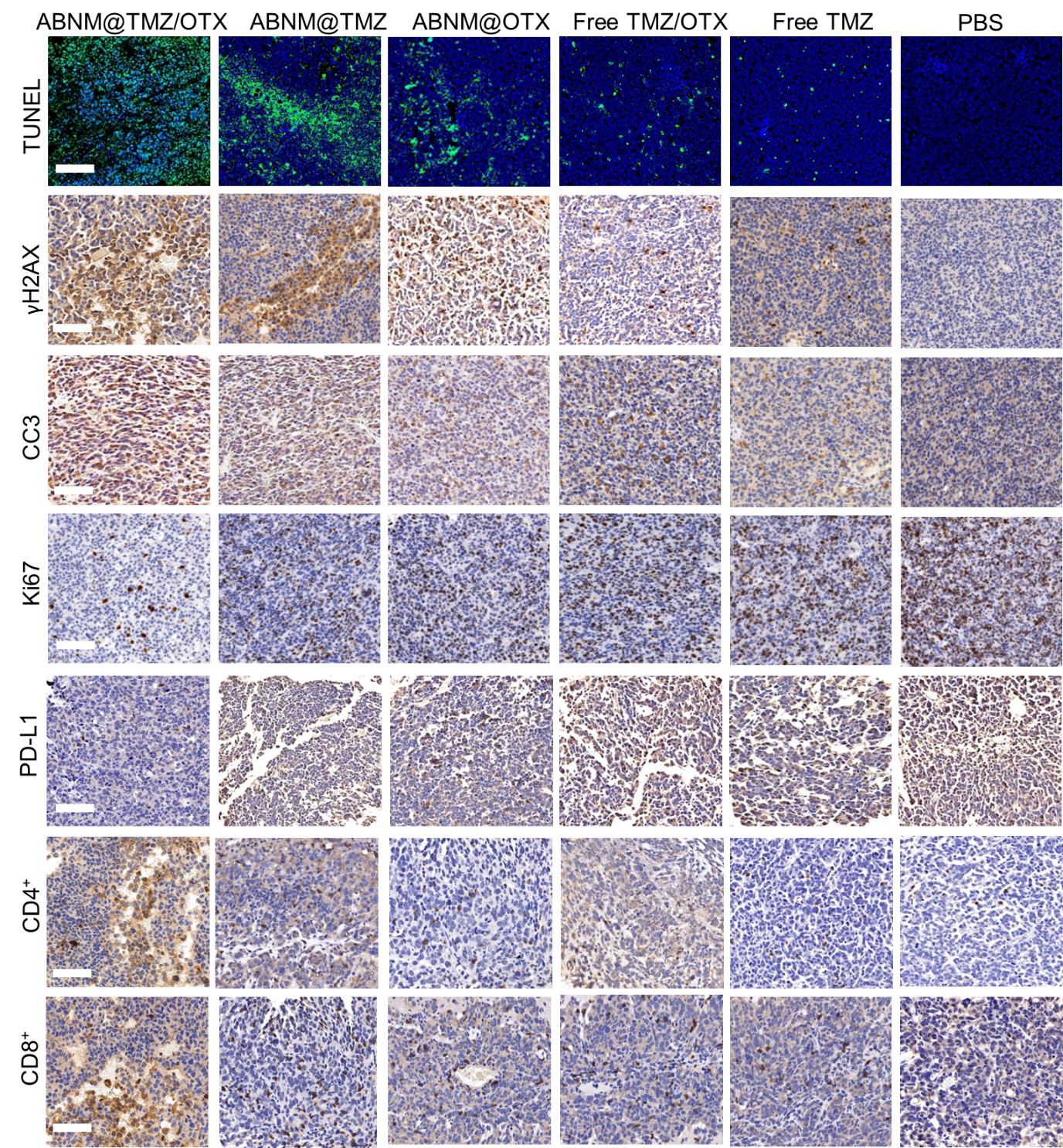


**Figure S12**. TUNEL assay and immunohistochemical staining for γH2AX, cleaved caspase 3 (CC3), proliferation (Ki67), PD-L1, CD4^+^ and CD8^+^ in tumor slices taken from mice treated with ABNM@TMZ/OTX, ABNM@TMZ, ABNM@OTX, free TMZ/OTX, free TMZ or PBS. Quantification of number of tumor cells that stained positive for markers of γH2AX (scale bar = 200 µm).


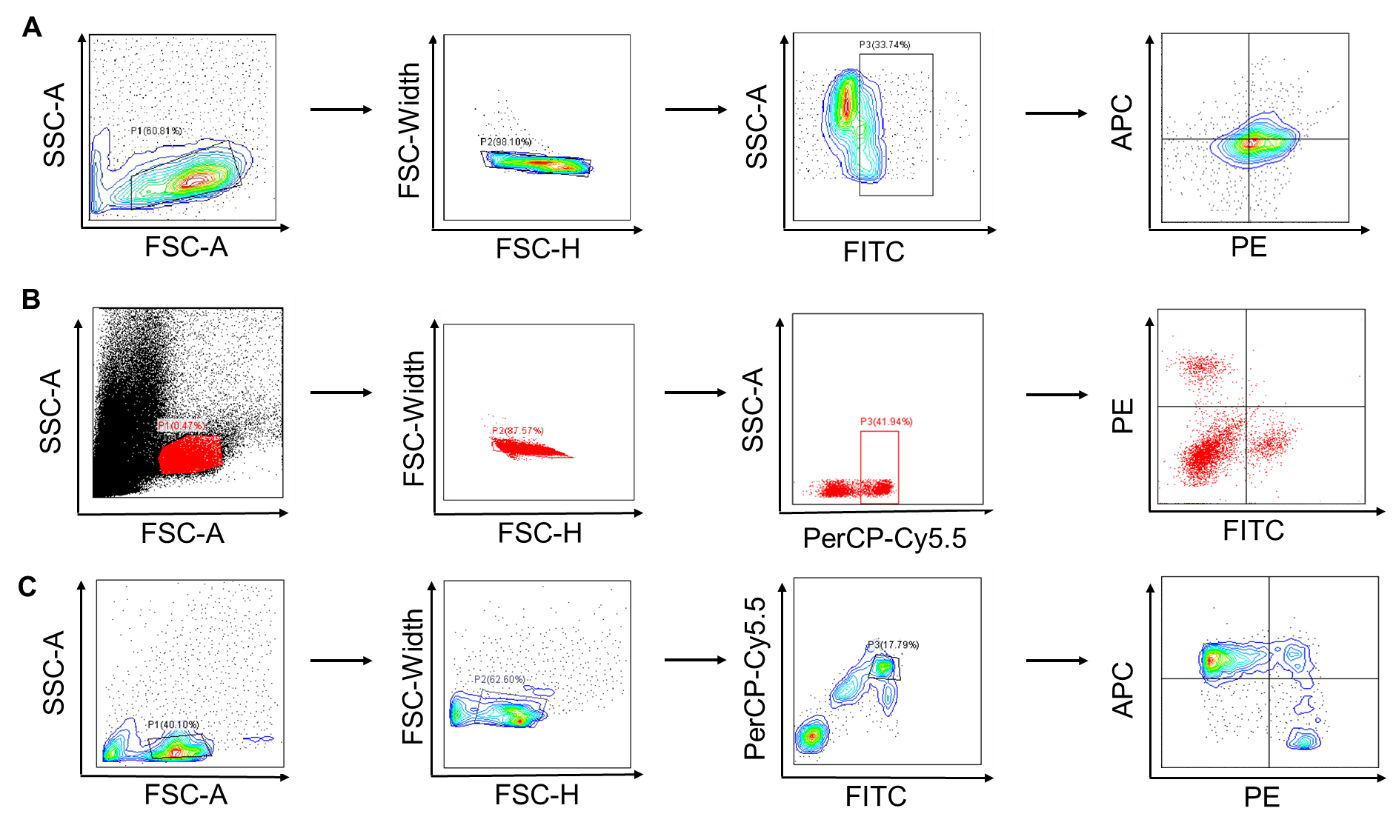


**Figure S13.** Flow cytometry gating strategy for the analysis of DC maturation **(A)**, CTL **(B)** and memory T cells **(C)** *in vivo*.

**References**

1. Y. Liu *et al.*, Charge conversional biomimetic nanocomplexes as a multifunctional platform for boosting orthotopic glioblastoma RNAi therapy. *Nano Lett.* **20**, 1637-1646 (2020).

2. Y. Zou *et al.*, Effective and targeted human orthotopic glioblastoma xenograft therapy via a multifunctional biomimetic nanomedicine. *Adv. Mater.* **30**, 1803717 (2018).

3. Y. Jiang, J. Zhang, F. Meng, Z. Zhong, Apolipoprotein E peptide-directed chimeric polymersomes mediate an ultrahigh-efficiency targeted protein therapy for glioblastoma. *ACS Nano* **12**, 11070-11079 (2018).
